# A Comprehensive Analysis of Multilayer Community Detection Algorithms for Application to EEG-Based Brain Networks

**DOI:** 10.3389/fnsys.2021.624183

**Published:** 2021-03-01

**Authors:** Maria Grazia Puxeddu, Manuela Petti, Laura Astolfi

**Affiliations:** ^1^Department of Computer, Control and Management Engineering “Antonio Ruberti”, University of Rome Sapienza, Rome, Italy; ^2^IRCCS Fondazione Santa Lucia, Rome, Italy

**Keywords:** community detection, network neuroscience, modularity, electroencephalography, statistical analysis

## Abstract

Modular organization is an emergent property of brain networks, responsible for shaping communication processes and underpinning brain functioning. Moreover, brain networks are intrinsically multilayer since their attributes can vary across time, subjects, frequency, or other domains. Identifying the modular structure in multilayer brain networks represents a gateway toward a deeper understanding of neural processes underlying cognition. Electroencephalographic (EEG) signals, thanks to their high temporal resolution, can give rise to multilayer networks able to follow the dynamics of brain activity. Despite this potential, the community organization has not yet been thoroughly investigated in brain networks estimated from EEG. Furthermore, at the state of the art, there is still no agreement about which algorithm is the most suitable to detect communities in multilayer brain networks, and a way to test and compare them all under a variety of conditions is lacking. In this work, we perform a comprehensive analysis of three algorithms at the state of the art for multilayer community detection (namely, genLouvain, DynMoga, and FacetNet) as compared with an approach based on the application of a single-layer clustering algorithm to each slice of the multilayer network. We test their ability to identify both steady and dynamic modular structures. We statistically evaluate their performances by means of *ad hoc* benchmark graphs characterized by properties covering a broad range of conditions in terms of graph density, number of clusters, noise level, and number of layers. The results of this simulation study aim to provide guidelines about the choice of the more appropriate algorithm according to the different properties of the brain network under examination. Finally, as a proof of concept, we show an application of the algorithms to real functional brain networks derived from EEG signals collected at rest with closed and open eyes. The test on real data provided results in agreement with the conclusions of the simulation study and confirmed the feasibility of multilayer analysis of EEG-based brain networks in both steady and dynamic conditions.

## Introduction

The convergence of networks science to neuroscience has opened the way to the currently well-established network neuroscience framework (Bassett and Sporns, 2017), an emerging field that aims to investigate brain organizational principles by means of networks science tools. This shift was driven by two aspects. On one side, the development of tools to investigate complex systems has exploded, as more and more complex data from different fields (i.e., social, transportation, and biological sciences) become available (Newman, 2003; Boccaletti et al., 2006). On the other side, the advancements in neuroimaging techniques led to consequent improvements in the field of brain connectivity (Jirsa and McIntosh, 2007), which allows modeling of brain structure and function as a result of complex networks of brain areas (nodes) anatomically or functionally interconnected (Sporns, 2011).

An emergent property of networks representing real complex systems is the community structure (Porter et al., 2009; Newman, 2012). A specific type of communities is the modules, groups of nodes densely connected which can be related to specific functions of the system and widely observed in brain networks (Meunier et al., 2010; Sporns and Betzel, 2016; Betzel, 2021). Previous studies pointed out how a modular structure represents a mean to reveal non-trivial relationships between topological and functional features of the complex networks (Guimerà and Amaral, 2005). This property of the brain network is located halfway between global and local scales, at a mesoscale level, which is informative of the network's organization (Betzel and Bassett, 2017). In fact, while at local and global scales the focus is on the fundamental units of the network (nodes) and on the network as a whole, at this intermediate scale, we can observe how the network's elements organize themselves, e.g., into clusters, to form efficient systems. In this sense, communities underpin the brain network's organization: their composition shapes the communication patterns of the system and promotes well-balanced and efficient mechanisms of integration and segregation between brain sub-systems (Betzel et al., 2013; Sporns, 2013; Wig, 2017).

While most of the studies on community detection in brain graphs deal with single-layer networks, especially in electroencephalographic (EEG) applications (Chavez et al., 2010; Ahmadlou and Adeli, 2011; Zippo et al., 2018), brain networks are intrinsically multilayer (Hutchison et al., 2013; Muldoon and Bassett, 2016; De Domenico, 2017). There is no single neuronal connectivity pattern able to fully represent brain functioning: rather, brain interactions vary across multiple domains. They evolve in time or according to the subject's conditions, the tasks, or the frequency span (in M/EEG acquisitions). Thus, a multilayer framework better accounts for the complexity and diversity of cerebral interactions, resulting suitable to analyze brain connectivity without either throwing away or combining different information.

A multilayer network is a sequence of linked single-layer networks, each one encoding specific attributes of the system. It allows the integration of multiple channels of connectivity to provide a more natural description of the brain system, as the nodes (brain areas) can show different sets of interactions at each layer. A particularly interesting case for EEG-based analysis is represented by time-varying multilayer networks. Being able to track the brain organization during a task or a cognitive state is of interest because changes, as well as steady states, of the network's structure could be physiologically meaningful. For this reason, it is worthwhile to investigate modular structure in brain networks, especially those reconstructed from EEG signal, which benefit from an excellent temporal resolution. Under this perspective, multilayer analysis of EEG-derived networks can be successfully used to gain insights in applications that require an accurate temporal resolution, like epilepsy, vision, or cognition (Zahra et al., 2017).

Recovering communities in a multilayer network is usually done algorithmically because of the real networks' usually big dimension and complexity. A range of algorithms have been proposed, spanning along three main approaches:

- The first one trivially consists of applying a single-layer clustering algorithm to each slice of the multilayer network. Previous comparative analysis (Lancichinetti and Fortunato, 2009) has highlighted the good performances of those based on modularity optimization (Girvan and Newman, 2002; Newman and Girvan, 2004). In particular, the one introduced in Leicht and Newman (2008), which, from now on, we will call ModStat (stationary modularity), showed good performances with directed EEG brain networks (Puxeddu et al., 2017).
- The second approach is based on the optimization of a multilayer formulation of modularity (Mucha et al., 2010). The implementation of this approach is provided in (Jeub et al., 2019) and is known as genLouvain. This algorithm represents an extension of the classical modularity maximization (Blondel et al., 2008), to which it adds a term that considers the coupling of the nodes across layers. This term is proportional to a resolution parameter, ω, which determines the stability of the network partitioning across the slices.
- The third approach consists of the optimization of a multi-objective function (Chakrabarti et al., 2006), which aims to maximize both the accuracy of the partitions at each layer and the smoothness across all the layers. Two widely used algorithms reflecting this last approach are DynMoga (Folino and Pizzuti, 2014) and FacetNet (Lin et al., 2008, 2009). The former is a genetic algorithm that optimizes modularity and mutual information of consecutive layers. The latter discovers communities iteratively, taking into account both the observed data and a probabilistic model given by all the single community structures.

To date, an agreement on which is the most advantageous approach is missing. In the recent years, some efforts have been made on investigating their behavior on multilayer networks. A conventionally used approach, even in single-layer network analysis, consists of testing the algorithms on a real network with a known community structure (Lancichinetti and Fortunato, 2009). In Silva et al. (2016), for example, the authors compared the behavior of algorithms based on evolutionary clustering on a high school network, the MIT Social Evolution dataset and the Brazilian Congress network, in which the ground truth is respectively represented by classes, dormitory sectors (Dong et al., 2011), and political alignment of the congressmen based on their party. However, this approach might lack generalization, and the obtained results would be limited to that specific network properties. Moreover, a brain network in which the community structure is known *a priori* does not exist. Hence, the lack of ground truth for brain communities, together with their ubiquity, requires the implementation of benchmark networks with a known community structure and realistic features to test different community detection algorithms. In Silva et al. (2016), the authors also tested the algorithms on a synthetic network. Nevertheless, it is a simple network with few nodes and three clusters that can hardly be encountered in neuroscience. In Schmidt et al. (2018), the authors tested two multilayer clustering approaches on an artificial network with more realistic properties. However, the test made on a single network, as previously said, might lack generalization of the results. Other already existing tools (Lin et al., 2008; Kim and Han, 2009) are a multilayer version of the Girvan and Newman model (Girvan and Newman, 2002) and do not allow a deep analysis of the algorithms, as they constrain most of the parameters characterizing the network (e.g., number of nodes, number of clusters, *etc*.). In Granell et al. (2015), the authors propose a tool in which a potential user can set some parameters of interest, such as the number of nodes, number of clusters, and ratio between intra-cluster and inter-cluster density. However, such tool does not address some aspects that are pivotal for EEG-based networks, like the noise level.

The principal aim of this work is to identify the most suitable approach to recover communities in EEG-based multilayer brain networks. For this purpose, we aim to perform a comparative analysis whose results will furnish practical guidelines about the use of multilayer community detection algorithms in the context of EEG-derived brain networks. Thus, we introduce a flexible toolbox able to generate artificial networks with a modular structure, with manifold features. This tool is a multilayer extension of the single-layer generator introduced in Puxeddu et al. (2017). The number of nodes, graph density, number of clusters, noise level in the community structure (modeled as a random permutation of a certain number of links), and percentage of nodes moving from a module to another one at a given layer can be set by the user. With respect to the previously described tools, we can also generate networks with different levels of noise to take into account the false positives and false negatives resulting from any brain functional connectivity estimation. In the case of EEG signals, the noise might depend on different factors, such as physiological/instrumental artifacts (Fisch, 1999; Riitta Hari and Aina Puce, 2017) and fluctuations in the EEG activity, or it may arise as a result of the connectivity estimation methods (Astolfi et al., 2007; He et al., 2019).

Using the proposed benchmark graphs, we performed a comparative analysis of the different multilayer clustering algorithms, testing them on graphs generated accounting for a wide range of network features systematically varied in the range typical of EEG-based brain networks. Furthermore, here for the first time we considered two scenarios: one in which the community structure is stationary across the layers and one in which it changes dynamically. Both cases are of great interest in real applications. In the first case, we aim to get a single partition out of a multilayer network with persistent organizational features. This is the case of layers associated to time points of stationary phenomena or to different subjects of the same category (e.g., healthy subjects or patients) for which we are interested in using the multilayer approach to extract enduring features. In the second case, we aim to track mesoscale organization in multilayer networks underlying non-stationary phenomena or different clinical cohorts. In both cases (stationary and evolving community structure), we statistically evaluated the algorithms' performances under different conditions by means of an analysis of variance (ANOVA).

Finally, as a proof of concept, we applied the four approaches to a brain functional multilayer network estimated from EEG signals acquired in a healthy subject during resting state at closed eyes and open eyes. We report the differences between the community structure subtending the two phases obtained by using the investigated algorithms, with the aim to test their accordance with the guidelines provided by the simulation studies. This application to real data has the purpose of validating the results of the simulation studies in a well-known and studied condition in order to check the applicability of multilayer community detection tools to EEG-based brain networks.

## Methods

### Benchmark Network Generation

The toolbox that we developed generates pseudo-random multilayer networks with a defined community structure and consists of an algorithm implemented in Matlab environment (release 2017b). A preliminary version of the toolbox was reported in Puxeddu et al. (2019). This toolbox allows a potential user to create networks with either stationary or evolving community structure with features spanning a variety of conditions experimentally observable in EEG-based brain networks. In the following paragraphs, we describe the implementation of the toolbox for each of the above-mentioned two cases.

#### Networks With Stationary Community Structure

**Figure 1:**
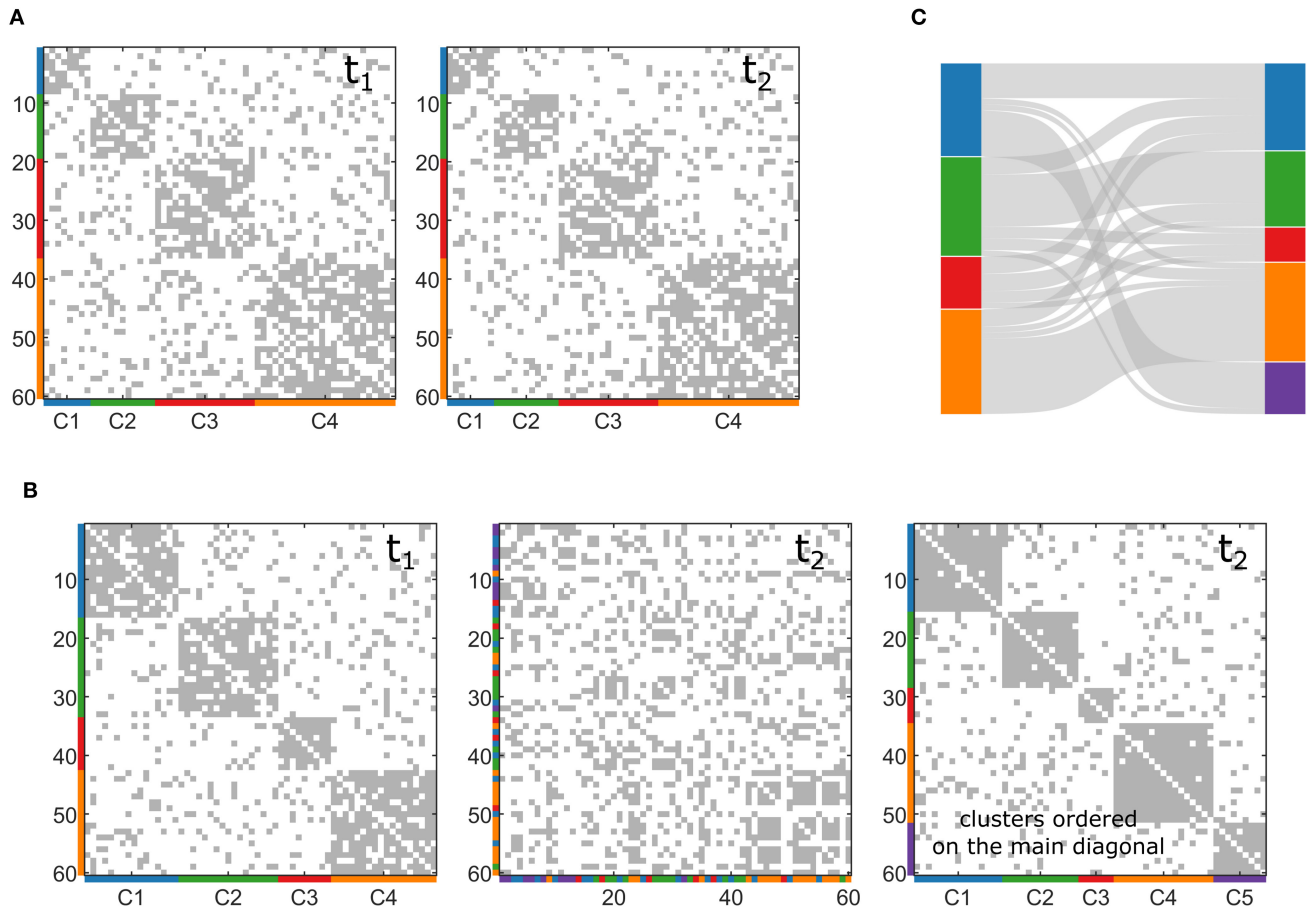
Examples of synthetic multilayer networks generated through the toolbox. **(A)** Two snapshots (t_1_ and t_2_) of a multilayer network with stationary community structure. **(B)** Two snapshots (t_1_ and t_2_) of a multilayer network with evolving community structure. In the second t_2_, the nodes are re-ordered to represent clusters on the main diagonal. **(C)** Sankey diagram of the network generated in **(B)**.

The network generated by the toolbox, in this case, presents a stationary modular structure, in which the composition of the clusters across the layers does not change. Here the variability between layers is only due to the noise level, which might make some links appear or disappear. Figure 1A shows an example of two layers of a multilayer network generated in this fashion. As mentioned before, the main advantage of this toolbox is its flexibility. In fact, the users can set several features which will characterize the network: number of nodes (*N*), graph density (*D*), number of clusters (CN), the ratio between intra-cluster and inter-cluster density (dr), the noise level (no), and the number of layers (nL). Once the set of desired features is selected, the algorithm proceeds by two main steps:

- Creation of a single-layer network (binary and directed) exploiting the algorithm described in Puxeddu et al. (2017)—we will use this network as a basis for each layer.
- Addition of the percentage of noise (i.e., percentage of links randomly shifted) set as input to each layer.

With these two steps, we obtain a multilayer network in which each slice has the same imposed community structure obtained in (a), and the inter-layer variability is only due to the presence of noise applied to each network (b). Step (a), in turn, consists of four stages:

(a.i) Setting of the size of the communities by randomly choosing CN integers, with the only constraint that their sum is equal to *N*.
(a.ii) Wiring of the network by randomly filling an *N* × *N* empty matrix observing the imposed specifics (about density and ratio between intra-cluster and inter-cluster density).
(a.iii) Checking the absence of isolated nodes inside the clusters, and if present, the algorithm rewires the intra-cluster connections.
(a.iv) Ensuring that the internal degree of each node is higher than the external degree (with respect to its cluster) by rewiring.

#### Networks With Evolving Community Structure

In this second case, we want our toolbox to simulate a multilayer network with a community structure that changes node composition across the layers. In this case, the algorithm in the toolbox also starts generating a first layer (with the same stages described above), but then it generates the following slice so that a certain percentage of nodes (pn, set as input by the user) changes its allegiances to modules. The algorithm acts only on the connections related to the nodes that change membership, maintaining the rest of the networks as it was originated at the beginning. Similarly, it can also increase or decrease the number of clusters, CN, moving some nodes into a new community or moving all the nodes belonging to one community in the remaining ones. In this way, the user can obtain controlled variations of different entities of the community structure according to the selected percentage of nodes that must change cluster (pn) and to the possible creation or disappearance of communities. Figure 1B reports an example of two layers of a multilayer network with changing community structure, in which pn has been set to 30% and the number of clusters increases with the appearance of a new one (in purple in the figure). We represent this dynamic community structure through the Sankey diagram in Figure 1C.

### Simulation Studies for the Algorithm Comparison

#### Stationary Community Structure

We made a simulation study testing the algorithms on benchmark networks with a stationary community structure generated as described in “section Networks With Stationary Community Structure.” We exploited the tool by systematically varying the network features represented by the input parameters. In particular, we explored a range of values for the parameters according to those experimentally met in EEG-based functional brain networks:

- *N* = 60
  We selected this value to mimic the 61-channel configuration typically used in most EEG studies.
- *D* = [0.10, 0.30]
  We simulated sparse networks with two different density levels in a range usually met with real data.
- CN = [2, 4, 6]
  We simulated different parsing of the network to have coarser as well as finer community structures.
- dr = 2
  We generated networks in which the intra-cluster density is twice with respect to the inter-cluster one. We do this in order to start from a very convenient condition for the algorithms that we will gradually deteriorate by adding different noise percentages.
- no = [10, 25, 50%]
  These noise percentages were chosen to reproduce networks with different levels of module clearness.
- nL = [2, 10, 50, 100]
  We consider networks with different numbers of layers to see if this factor influences the algorithms' performance. Indeed we expect multilayer algorithms exploiting a higher dimensionality to mitigate the noise effect.

**Figure 2:**
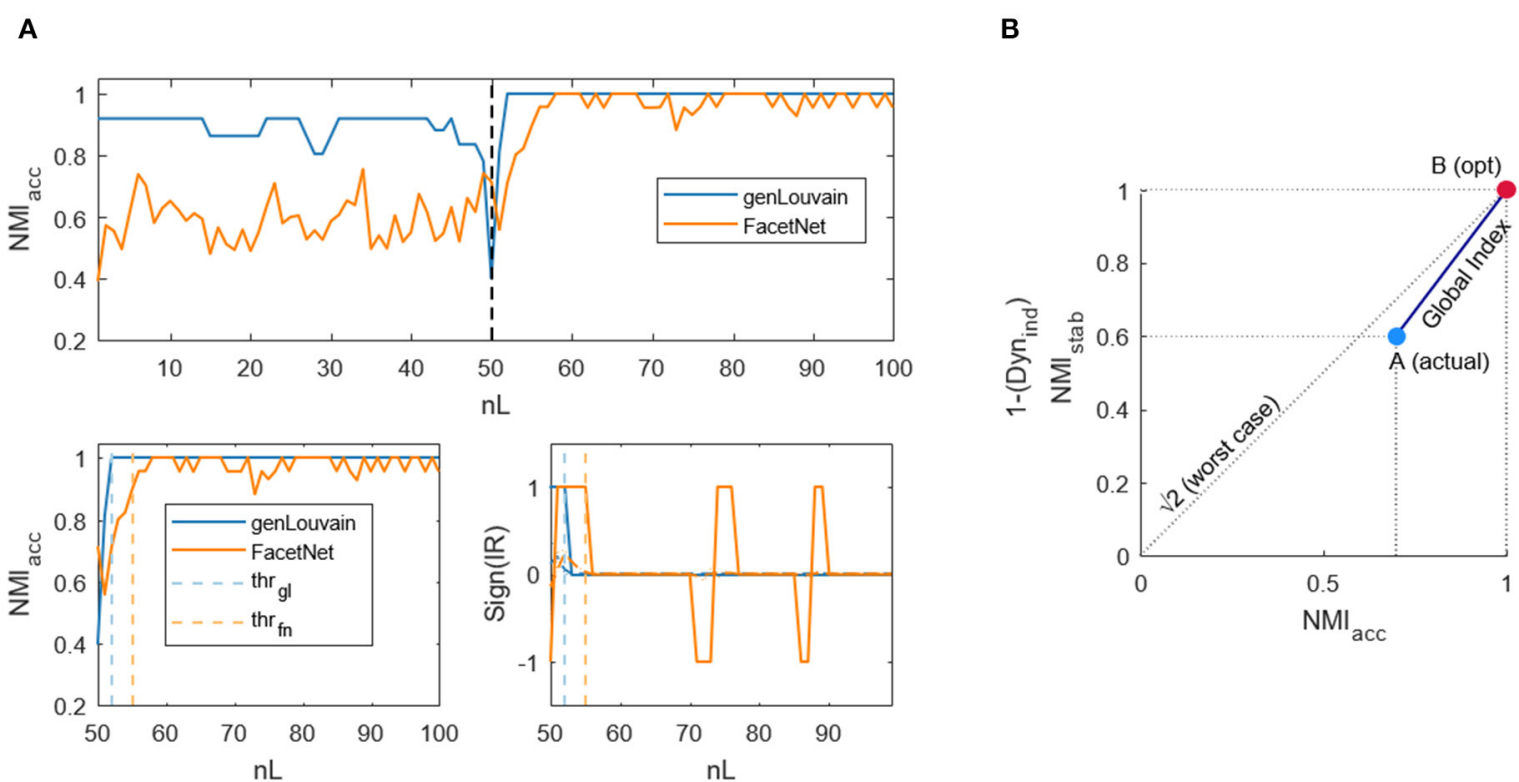
Example of dynamic and global indices computation. **(A)** Dynamic index. Top figure: normalized mutual information computed between the output of the algorithms genLouvain and FacetNet and the actual community structure of a generated network with 100 layers. Lower left figure: normalized mutual information from the snapshot in which community structure changes and threshold samples (from which the algorithms go to regime) identified through the dynamic index. Lower right figure: sign of the first derivative smoothed and threshold samples. **(B)** The global index is indicated with the dark blue continuous line. A is the point corresponding to the actual values of NMI_acc_ and NMI_stab_/Dyn_ind_, while B is the point corresponding to the maximum values reachable by the indices.

Then, we run the four algorithms (genLouvain, ModStat, DynMoga, and FacetNet). To evaluate the effect of the factors algorithm, number of clusters, noise level, and number of layers, we performed a repeated-measure ANOVA using three figures of merit as dependent variables in order to capture different aspects of the performance:

- Accuracy: To evaluate the algorithms' accuracy, we used the normalized mutual information (NMI) (Danon et al., 2005). This is an index borrowed from the field of information theory and used to estimate the similarity between two objects. It can range between 0 (completely different objects) and 1 (identical objects). It has been already employed in this context to calculate the similarity between two given partitions that, in our case, are the ones obtained from the clustering algorithms and the known community structure. We computed the NMI between these two partitions in each layer, and then we used the average of all these values as index of accuracy. We will refer to this index as NMI_acc_.
- Stability: In networks with stationary community structure, it is also important to assess how much the clustering algorithms provide for a stable partition across all the layers. Thus, we computed the NMI between each layer and the following one, and we computed the average of these values to obtain an index of stability. We named this index NMI_stab_.
- Global performance. We finally wanted an index summarizing the global performances of the algorithms, simultaneously considering accuracy and stability. We computed this index as the Euclidean distance between two points, A and B, in the xy plane where the x and y axes represent, respectively, the values of accuracy and stability. A is the point [x_(acc)_, y_(stab)_] associated to the actual values of accuracy and stability assumed by the algorithm, and B is the point [1, 1] that represents the optimum (both stability and accuracy reach their highest score, which is 1). In this way, the Euclidean distance between A and B, which we used as index of global performance, represents the distance of the algorithms' performance from the optimal one. An example of this index is shown in Figure 2B. We will refer to this index as GS_ind_, and it varies between 0 (optimal performances, A = B) and $\sqrt{2}$ (worst performances, NMI_acc_ = NMI_stab_ = 0, A is the point [0, 0] in the xy plane).

Since the algorithms genLouvain and FacetNet depend on the inter-layer resolution parameters ω and λ, we made two preliminary analyses exploring the behavior of the algorithms under different values of these parameters in order to select the best possible values of ω and λ for the stationary condition to be used in the comparative analysis. For this purpose, we performed two more ANOVA tests for repeated measures, one for genLouvain and one for FacetNet, considering values of ω and λ in the range [0.1, 10] and [0.1, 1], respectively. The first study was aimed at evaluating the effect of the factors ω (levels: 0.1, 0.2, 0.5, 1, 2, 5, 10), cluster number, noise level, and number of layers on the performance of genLouvain. Similarly, the second one was meant to evaluate the effect of λ (levels: 0.1, 0.2, 0.5, 0.7, 0.8, 0.9, 1), cluster number, noise level, and number of layers on the performance of FacetNet. The results of these two analyses are detailed in the Supplementary Material, sections 1 and 2, and have been used in the main comparative analysis to run the two algorithms with the appropriate choice of ω and λ according to the network's features.

#### Evolving Community Structure

To generate benchmark networks with dynamic community structure, we exploited the toolbox in the version introduced in “section Networks With Evolving Community Structure.” We generated the networks by setting the input parameters to the same values reported in “section Stationary Community Structure,” but here we also included the parameter pn (percentage of nodes changing allegiance to modules) with the following values, chosen to simulate progressive variations of the community composition: 10, 30, 50, 70, and 100%.

The resulting networks present a variation only between the first and the second half of the layers, while within the two halves the community structure is stationary, to simulate the transition between two different tasks or two classes of subjects (e.g., healthy subjects vs. patients). We run the four algorithms again, and we performed an ANOVA for repeated measures using, as dependent variables, three different indices to capture different aspects of the performances:

- Accuracy: To evaluate the algorithms' accuracy, we used the normalized mutual information (NMI_acc_) defined as in “section Stationary Community Structure.”
- Dynamics: In networks with evolving community structure, it is also important to assess the rapidity with which the algorithms recognize the variation of the modules' composition. Thus, we defined and implemented an index that points out how much it takes for the algorithms, in terms of number of layers, to exactly detect the new structure. The index mathematically identifies the layer (*l*_thr_) from which the NMI_acc_ (Figure 2A, upper panel)—which decreases in proximity of nL/2, where the community structure changes—becomes stable and enters a sort of plateau after the transition (Figure 2A, lower left panel). The idea is that the incremental ratio (IR) of the NMI_acc_ curve from nL/2 to nL will be positive until the algorithm goes to regime and null from that point on. Thus, we computed the IR, we smoothed it to avoid spurious peaks due to the noise, and we considered the sign to capture when it becomes zero (Figure 2A, lower right panel). We find the threshold layer through the formula:
  (1) $$\begin{array}{l} l_{\text{thr}}\in \left[\frac{\text{nL}}{2}+1,\;\text{nL}\right]:=\begin{array}{l} \arg\max\\ l_{\text{thr}} \end{array}\left(\frac{\sum_{l=\frac{\text{nL}}{2}+1}^{l_{\text{thr}}}\text{sign}({\text{IR}}_{\text{smoothed}})}{\sum_{l=l_{\text{thr}}+1}^{\text{nL}}\text{sign}({\text{IR}}_{\text{smoothed}})}\right) \end{array}$$
  It scans all the layers from nL/2 +1 to *nL*, and for each *l* it computes the ratio between the sum of this function sign(IR_smoothed_) before and after *l*. Then, it takes as threshold the *l*_thr_ to which the maximum of this ratio corresponds. Ideally, at *l*_thr_, the numerator is positive (i.e., before *l*_thr_, the trend of NMI_acc_ is ascendant), and the denominator is equal to 0 (i.e., after *l*_thr_, the trend of NMI_acc_ is stable), so that the argument is infinite—the maximum possible. Once *l*_thr_ is obtained, we normalized it for nL/2 to obtain an index that varies in the range [0, 1], independently of the values of nL considered. We will refer to this index as to Dyn_ind_. The lower it is, the fastest are the algorithms in recovering the structure modification.
- Global performance: In analogy to the previous analysis, we computed an index that summarizes the global performances of the algorithms, considering at the same time accuracy and dynamics. It is computed as explained in “section Stationary Community Structure,” but here, instead of NMI_stab_, we consider the complement to unity of Dyn_ind_. We will refer to this index as GD_ind_.

In the case of evolving communities also, we performed a preliminary analysis to determine the optimal setting of the parameters ω and λ for the algorithms genLouvain and FacetNet to be used in the comparative analysis. The results of this test can be found in the Supplementary Material, sections 1 and 2. It is worth to note that the values of ω and λ selected for the evolving community structure are different from those resulting from the study on stationary community structure.

### Multilayer Community Detection on Rest CE/OE EEG Brain Networks

For the purpose of validating the results of the simulation studies, we tested the algorithms in real EEG brain networks with features analogous to those investigated in the simulations, relative to a simple and controlled condition.

EEG data have been recorded and amplified by a commercial EEG system (BrainAmp, Brainproducts GmbH, Germany) using 61 electrodes (according to the extended 10–20 International System), with reference attached to the forehead and sampling frequency of 250 Hz, in a healthy subject (female, 33 years old) during rest with closed eyes (CE) and open eyes (OE). The subject gave informed consent prior to her participation, and the experiment was approved by the local ethics committee before the data acquisition started. Data were acquired at the Neuroelectrical Imaging and BCI Laboratory at IRCCS Fondazione Santa Lucia in Rome. The session was composed of 26 trials of 200 s each. In the first 100 s, the subject was asked to keep her eyes closed (task 1—CE), while in the last 100 s she was asked to keep her eyes open (task 2—OE). We pre-processed the data through band-pass filtering (1–45 Hz) and segmentation in 2-s epochs. The data were visually inspected to exclude the presence of artifacts. For each segment, we estimated brain functional connectivity through partial directed coherence (Baccalá and Sameshima, 2001; Astolfi et al., 2006), a spectral estimator based on Granger causality which provides an estimation of the network for each frequency point. We then mediated the estimations in four EEG frequency bands, defined according to individual alpha frequency (IAF) (Klimesch, 1999) (IAF = 10 Hz), focusing in the alpha range (IAF-2, IAF+2), as of interest for resting state (Karbowski, 1990; Niedermeyer, 1997; Compston, 2010). We assessed the significance of the connections through the asymptotic statistics (Takahashi et al., 2007; Toppi et al., 2016).

For each of the two tasks, we obtained 50 {200 s/[2 s (epoch) ^*^ 2 (tasks)]} binary networks of dimension 61 × 61. Then, we selected nL/2 layers from task 1 (CE) and nL/2 from task 2 (OE) and concatenated them so as to obtain four multilayer networks under different conditions of nL, like in the simulations. The obtained networks were sized 61ch^*^61ch^*^(2, 10, 50, 100) nL. Finally, we run all the algorithms 100 times on the four multilayer networks to take into account their stochastic nature, which implies that they might provide (slightly) different partitions even if applied to the same network. In the simulation studies, this issue was addressed as we perform an ANOVA test for repeated measures, which implies that for each combination of the parameters we compute the community detection several times.

## Results

### Simulation Studies for Algorithm Comparison

#### Algorithm Comparison in Networks With Stationary Community Structure

In Table 1, we reported the results of the ANOVA comparative analysis made by exploiting simulated multilayer networks with stationary community structure and graph density equal to 0.3. Analogous results have been obtained, setting the graph density to the lower level, *D* = 0.1, and this can be found in the Supplementary Material, section 4.

**Table 1:** Results of the ANOVA test executed for the comparative analysis on networks with stationary community structure and graph density equal to 0.3.

	**NMI**_****acc****_				**NMI**_****stab****_				**GS**_****ind****_			
	***dof*(b)**	***dof*(w)**	***F***	***p***	***dof*(b)**	***dof*(w)**	***F***	***p***	***dof*(b)**	***dof*(w)**	***F***	***p***
Alg	3	891	5393.2	<10^−4^	2	594	13096	<10^−4^	3	891	10549	<10^−4^
No	2	594	9110.4	<10^−4^	2	594	7458.2	<10^−4^	2	594	9392.2	<10^−4^
nL	3	891	1481	<10^−4^	3	891	129.87	<10^−4^	3	891	1427.1	<10^−4^
CN	2	297	473.31	<10^−4^	2	297	163.81	<10^−4^	2	297	420.19	<10^−4^
Alg*no	6	1782	844.84	<10^−4^	4	1188	2544.1	<10^−4^	6	1782	1752.3	<10^−4^
Alg*nL	9	2673	476.30	<10^−4^	6	1782	54.923	<10^−4^	9	2673	392.78	<10^−4^
Alg*CN	6	891	75.997	<10^−4^	4	594	580.84	<10^−4^	6	891	28.266	<10^−4^
Alg*no*nL	18	5346	163.8	<10^−4^	12	3564	5.6138	<10^−4^	18	5346	143.14	<10^−4^
Alg*no*CN	12	1782	115.89	<10^−4^	8	1188	174.06	<10^−4^	12	1782	170.10	<10^−4^
Alg*nL*CN	18	2673	56.64	<10^−4^	12	1782	14.881	<10^−4^	18	2673	51.239	<10^−4^
Alg*no*nL*CN	36	5346	36.189	<10^−4^	24	3564	3.1725	<10^−4^	36	5346	29.976	<10^−4^

*For each considered index (dependent variables of the test) we report the degrees of freedom (dof), F, and p-values relative to single factors and the interactions among them*.

**Figure 3:**
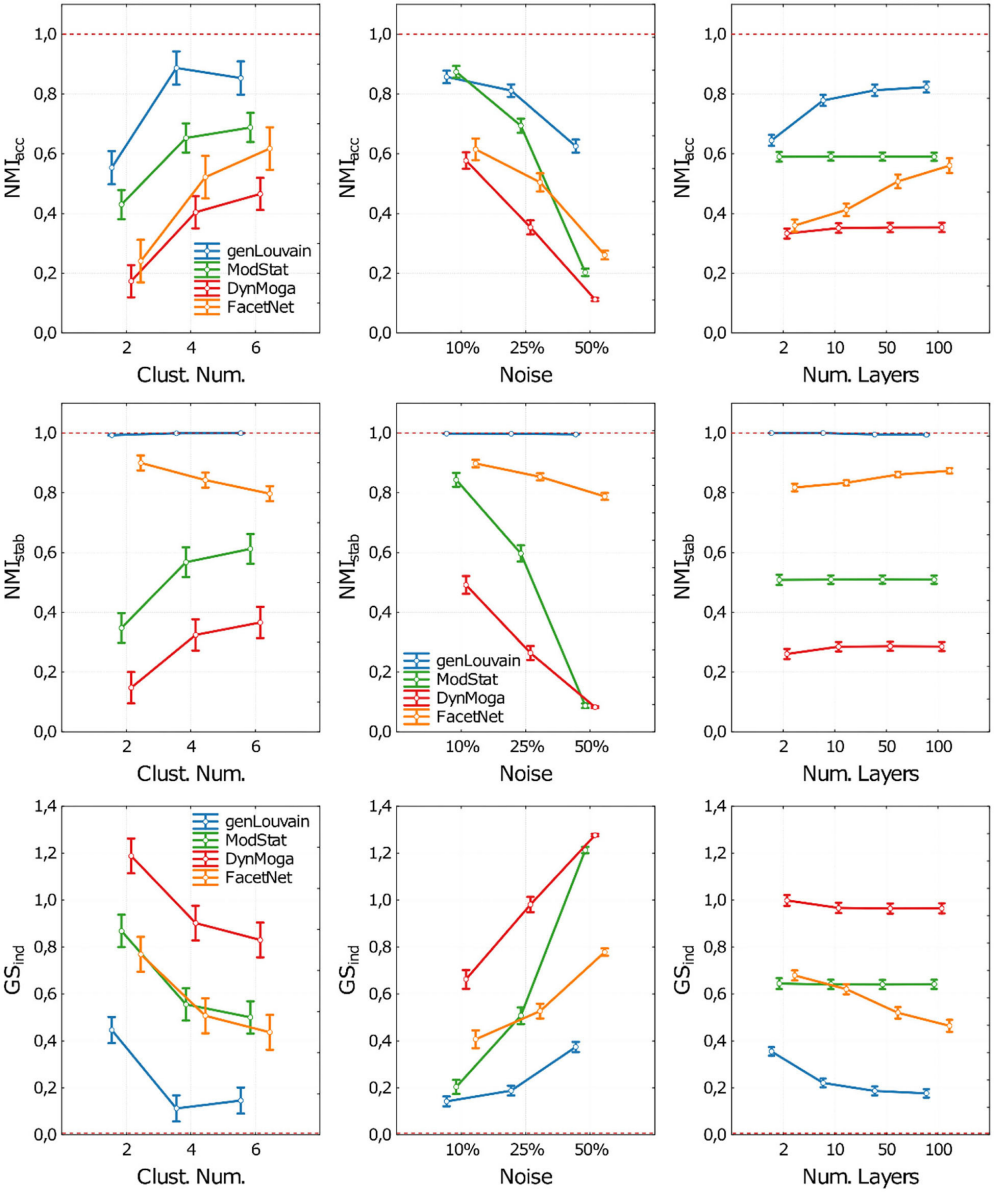
Plot of means and standard deviations of the three indices used to execute the comparative analysis on networks with stationary community structure. Each row of the figure corresponds to one index (NMI_acc_, NMI_stab_, GS_ind_). For each index we report three panels where we show the algorithms' performances with respect to the Clusters Number (first column), Noise level (second column), and number of network's layer (third column). Algorithms are identified through a color code (blue-genLouvain, green-ModStat, red-DynMoga, orange-FacetNet). In each panel we can see how the performance of the algorithms varies according the values the ANOVA factors and which algorithm reaches highest performances, in terms of accuracy (NMI_acc_), stability (NMI_stab_) or both (GS_ind_). The optimal performances are indicated though a red dotted line.

The related plot of means are reported in Figure 3, where the performances of the algorithms in terms of accuracy (NMI_acc_), stability (NMI_stab_), and both (*GS*_*ind*_) are shown as the number of clusters (CN), the level of noise (*n*), and the number of layers (nL) changed. For the sake of clarity, in each panel of Figure 3, we report the performances of the algorithms with respect to one factor, irrespective of the other two. In the Supplementary Material, section 3, the same results are reported extensively, and we can observe algorithm performances for each combination of the three ANOVA factors.

As for the accuracy (Figure 3, first row), all the algorithms have performance that is inversely proportional to the level of noise and directly proportional to the number of clusters simulated in the network. However, in noisy networks (no = 50%), genLouvain and FacetNet show an improvement of accuracy as the number of layers increases, above all if CN >2. In particular, genLouvain reaches almost the same level of accuracy in noisy and non-noisy networks, if nL ≥ 10 (see Supplementary Figure 9). On the contrary, as expected, the accuracy of ModStat is not affected by the number of layers, as it considers each slice of the network independently. Compared with the other algorithms, genLouvain displays a high level of accuracy in most combinations of noise, cluster number, and number of layers. The only exceptions are the case of low cluster number and low noise [CN = 2, no = 10%, nL = (2, 10, 50, 100)] in which ModStat has higher NMI_acc_ values for every value of nL.

Regarding the analysis of stability (Figure 3, second row), namely, the algorithms' capability to recover a stable partition across the layers of the network, the algorithm with the highest performance is genLouvain for each combination of the ANOVA factors. In fact, it always reaches the optimal value of NMI_stab_ despite the level of noise, number of clusters, and number of layers. For this reason, in this case, we excluded it from the ANOVA, as its NMI_stab_ distribution is not normal. On the contrary, the other algorithms are more sensitive to the ANOVA factors, especially to the level of noise and the number of clusters. The algorithm ModStat shows high values of NMI_stab_ (close to 1) in networks with low noise (no = 10%), while its performances decrease with higher noise levels. Overall, FacetNet displays high performances, with NMIstab >0.8 for each combination of the factors, while for DynMoga, the results show NMI_stab_ < 0.6 in every condition.

The evaluation of the global performances summarizes what is observed so far (Figure 3, third row).

In general, the results of ANOVA together with Tukey's *post-hoc* tests show all the algorithms having significantly higher performances in networks with low level of noise and high number of clusters. Overall, the figures show genLouvain outperforming the other algorithms.

#### Algorithm Comparison in Networks With Evolving Community Structure

In Table 2, we report the results of the comparative analysis made to test the algorithms on multilayer networks with evolving community structure, with density equal to 0.3 and cluster numbers unchanged. We observed analogous results in networks with lower density, *D* = 0.1, and increasing/decreasing cluster numbers, and we report them in the Supplementary Material, sections 4 and 5.

**Table 2:** Results of the ANOVA test executed for the comparative analysis on networks with evolving community structure and graph density equal to 0.3.

	**NMI**_****acc****_				**Dyn**_****ind****_				**GD**_****ind****_			
	***dof(b)***	***dof(w)***	***F***	***p***	***dof(b)***	***dof(w)***	***F***	***p***	***dof(b)***	***dof(w)***	***F***	***p***
**Alg**	3	2241	122200	<10^−4^	2	1494	2932.5	<10^−4^	2	1494	36095	<10^−4^
**No**	2	1494	255900	<10^−4^	2	1494	255.81	<10^−4^	2	1494	23219	<10^−4^
**nL**	3	2241	36813	<10^−4^	2	1494	2577.4	<10^−4^	2	1494	6071.1	<10^−4^
**p**	4	2988	37.25	<10^−4^	4	2988	7.81	<10^−4^	4	2988	16.17	<10^−4^
**CN**	2	747	14392	<10^−4^	2	747	477.02	<10^−4^	2	747	11828	<10^−4^
Alg*no	6	4482	18575	<10^−4^	4	2988	162.4	<10^−4^	4	2988	347.9	<10^−4^
Alg*nL	9	6723	12252	<10^−4^	4	2988	101.44	<10^−4^	4	2988	946.99	<10^−4^
Alg*p	12	8964	289.11	<10^−4^	8	5976	4.32	<10^−4^	8	5976	6.81	<10^−4^
Alg*CN	6	2241	425.4	<10^−4^	4	1494	341.71	<10^−4^	4	1494	475.52	<10^−4^
Alg*no*nL	18	13446	4514.8	<10^−4^	8	5976	7.11	<10^−4^	8	5976	91.28	<10^−4^
Alg*no*p	24	17928	46.64	<10^−4^	16	11952	2.31	0.002	16	11952	5.43	<10^−4^
Alg*no*CN	12	4482	3301.2	<10^−4^	8	2988	14.24	<10^−4^	8	2988	224.63	<10^−4^
Alg*nL*p	36	26892	33.27	<10^−4^	16	11952	3.25	<10^−4^	16	11952	1.19	*0.262*
Alg*nL*CN	18	6723	856.5	<10^−4^	8	2988	17.01	<10^−4^	8	2988	5.56	<10^−4^
Alg*p*CN	24	8964	308.26	<10^−4^	16	5976	6.56	<10^−4^	16	5976	13.88	<10^−4^
Alg*no*nL*CN*p	144	53784	44.58	<10^−4^	64	23904	1.41	0.017	64	23904	1.99	<10^−4^

*For each considered index (dependent variables of the test) we report the degrees of freedom (dof), F, and p-values relative to single factors and the interactions among them. Statistically non significant values have been reported with italic characters*.

**Figure 4:**
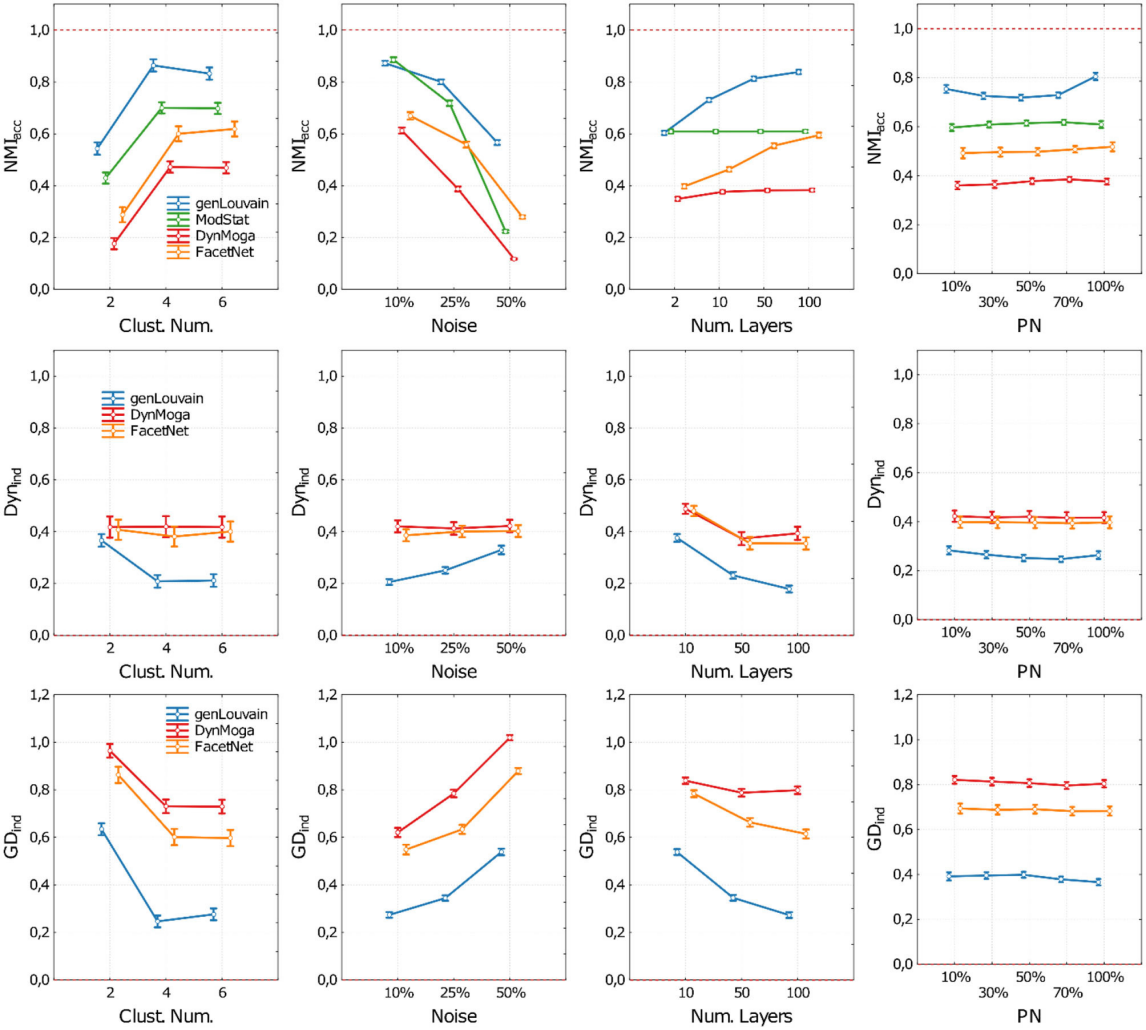
Plot of means and standard deviations of the three indices used to execute the comparative analysis on networks with evolving community structure. Each row of the figure corresponds to one index (NMI_acc_, Dyn_ind_, and GD_ind_). For each index, we report four panels where we show the algorithms' performances with respect to the cluster number (first column), noise level (second column), number of network's layer (third column), and percentage of nodes changing module (fourth column). Algorithms are identified through a color code (blue—genLouvain, green—ModStat, red—DynMoga, orange—FacetNet). In each panel, we can see how the performance of the algorithms varies according to the values of the ANOVA factors and which algorithm reaches highest performances in terms of accuracy (NMI_acc_), stability (Dyn_ind_), or both (GD_ind_). The optimal performances are indicated though a red dotted line.

In Figure 4, we represent the performances of the algorithms in terms of accuracy (NMI_acc_), dynamics (Dyn_ind_), and both (GD_ind_) as a function of the number of clusters (CN), the level of noise (no), the number of layers (nL), and the percentage of nodes changing modules (*p*) change. As in the previous study, to have more clear and informative representation of the results, in each panel of Figure 4, we report the performances of the algorithms with respect to one factor, irrespective of the other three. In the Supplementary Material, section 3, we reported the extensive results.

Regarding the accuracy, we show in the first row of Figure 4 the behavior of the algorithms with different levels of noise and number of layers. With a low level of noise, all the algorithms show a high accuracy in terms of NMI_acc_, regardless of the number of layers, while as the noise increases, there is a loss of accuracy. However, if nL ≥ 10, both genLouvain and FacetNet have a significant improvement of accuracy. All the algorithms are more accurate when applied on networks with CN ≥ 2, above all if nL ≥ 10. The percentage of nodes that change allegiance to modules does not substantially affect the accuracy of the algorithms. However, FacetNet and DynMoga show a little increase of performances when pn increases (see also Supplementary Figure 11), meaning that they can easily detect big changes. Overall, genLouvain has the highest NMI_acc_ values for each combination of the factors under analysis. The only exception is when CN = 2 and nL = 2, in which ModStat shows higher NMI_acc_ values.

As for the evaluation of the algorithm's dynamic (Figure 4, second row), we only considered the performances of genLouvain, DynMoga, and FacetNet. Considering also ModStat would not be meaningful, as it addresses each layer independently. Moreover, we considered only values of nL ≥ 2. GenLouvain displays the lowest Dyn_ind_ values for each combination of the factors under analysis, no, nL, CN, and *p*, meaning that it is the fastest in identifying changes of the community structure. Overall, the rapidity of the algorithms is directly proportional to the number of layers and the number of clusters while being inversely proportional to the noise level.

Finally, the global index (Figure 4, third row) confirms what was shown with the previous indices. It suggests that the factors that have the greatest influence on the algorithms' performances are the level of noise and the number of layers: an increase of their value provokes, respectively, a breakdown and a boost of the performances. The number of clusters is also proportional to the algorithms' performances, while the percentage of nodes that change a community does not substantially affect their behavior. The most sensitive to the network's features is genLouvain, which, in the comparative analysis, is the outperforming one, while DynMoga is globally the less sensitive.

The results of ANOVA together with Tukey's *post-hoc* tests show all the algorithms having significantly higher performances in networks with a low level of noise and a high number of clusters. In reverse, the factor percentage of nodes moved (pn) does not dramatically affect the global performances of the algorithms under analysis, meaning that the algorithms can detect small as well as big changes in community structure.

### Multilayer Community Detection on Rest CE/OE EEG Brain Networks

**Figure 5:**
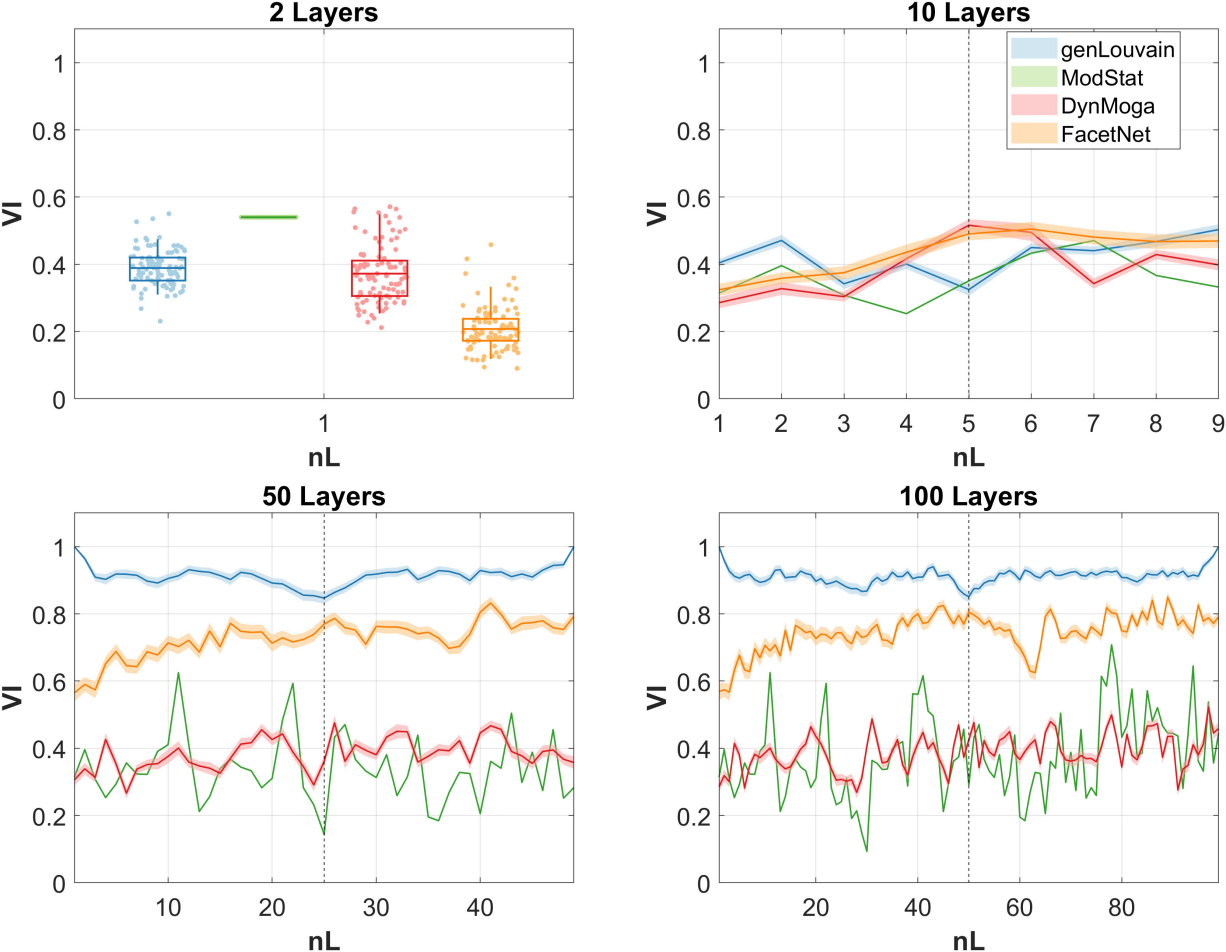
Normalized mutual information (NMI) computed between the output of the algorithms, identified with a color code, at consecutive layers of the multilayer network. As we run the algorithms 100 times, we report the means of the NMI at each snapshot, bounded by the confidence interval, represented with a lighter color. Each graph corresponds to one of the four networks extracted with different numbers of layers.

In this section, we present the results of the application of the four algorithms under analysis to EEG networks subtending CE and OE resting state in alpha band. In Figure 5, we report the trend of the normalized mutual information computed between the output of the algorithms across consecutive layers for all the estimated networks with nL = (2, 10, 50, 100). The black dashed line divides the CE state from the OE. Ideally, one would expect high and stable values of NMI in the two halves and a collapse of the index near to the dashed line. That would mean that the algorithm is able to extract two steady partitions in the two conditions which are different from each other and to detect the transition. In the case of nL = 2 instead, a value of NMI inferior to 1 is desirable, hopefully low. In line with the simulation study, genLouvain, with the resolution parameter set through the guidelines given by the preliminary analysis, is the algorithm that better approximates this behavior. Both genLouvain and FacetNet show higher stability and maximum discriminability between the two conditions when the number of layers increases. As also proven in the previous section, between the two, FacetNet results to be slower in detecting the change between the two tasks, and within each task, it is less stable and thus less accurate in detecting the community structure during CE or OE. DynMoga shows a mild increment of performance with a higher number of layers, even if they are lower compared to the other algorithms. Conversely, ModStat behaves independently from the number of layers, as it works on a single-layer level.

**Figure 6:**
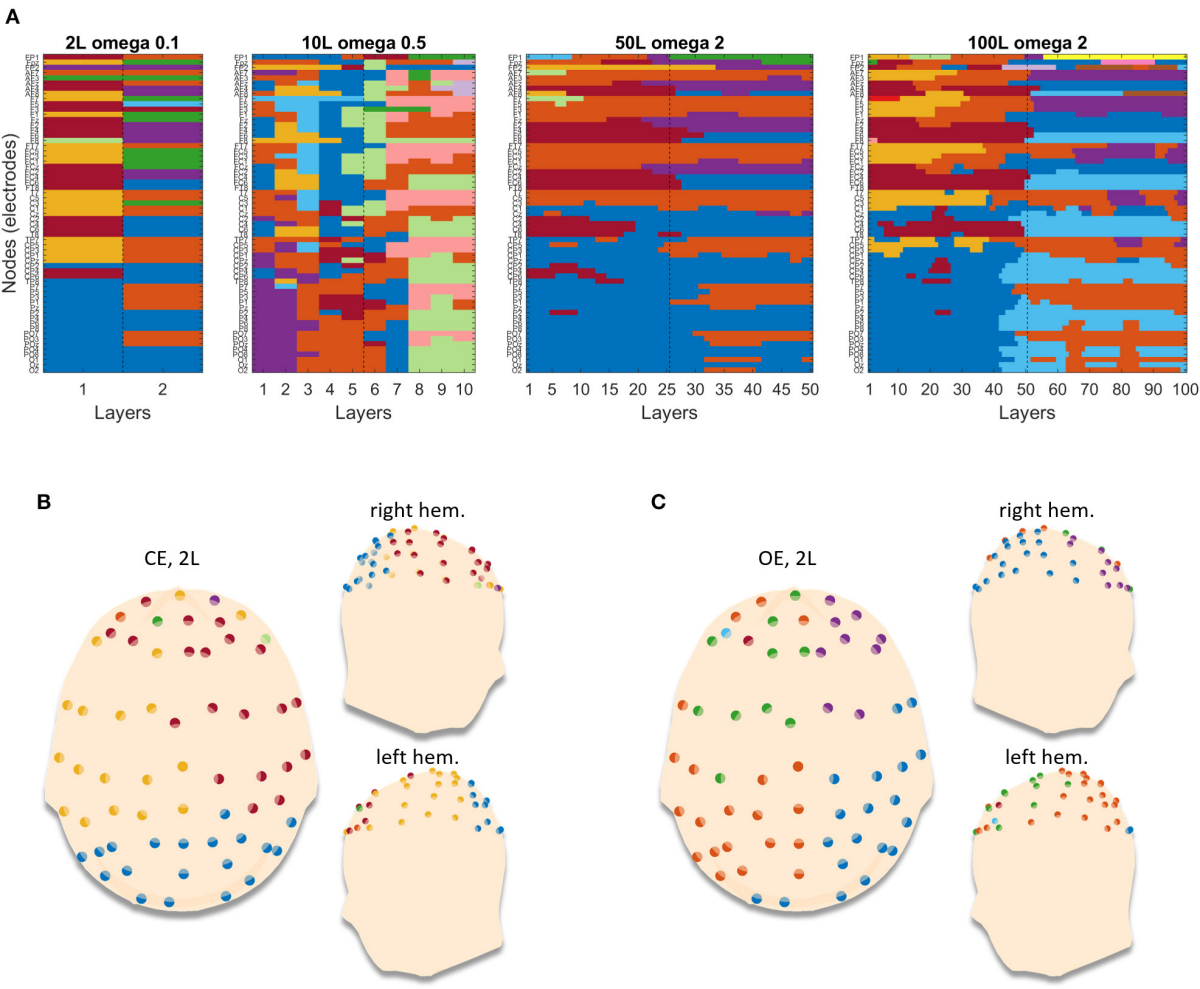
Example of partitions obtained by running genLouvain on the EEG brain networks. **(A)** The four images stand for the four networks with different numbers of layers. Each image has on the y-axis the nodes (channels) and on the x-axis the layers, and the cluster's membership is represented through colors. **(B,C)** Reported projections of the detected communities on a 3D model of scalp for the two conditions, closed eyes and open eyes, respectively. In each panel, the 3D model is seen from above, with the nose pointing to the upper side of the page, and laterally. The dots are the 61 electrodes grouped into clusters and displayed with different colors.

We finally show in Figure 6A how these multilayer networks are parsed in clusters by genLouvain, which is the most advisable algorithm after our simulations. The figure reports, as representative, one of the 100 repetitions computed which, as indicated by the narrow confidence interval in Figure 5, are very much similar among them. The partitions are consistent across all the levels of nL, and in Figures 6B,C, we show the partitioning of the network for each condition, CE and OE in the case in which nL = 50. During the CE phase, there is a cluster that involves the occipital electrodes and two clusters composed by electrodes from the left and the right hemisphere, respectively. During the OE phase, the first cluster is dismembered between the left and the right hemispheres, and one can observe the modules becoming more hemisphere specific. Such results are observed both in the EEG network made of nL = 2 and in the ones with nL > 2, with different ω*-*values properly chosen according to the preliminary analysis (Supplementary Material, section 1).

## Discussion

This work aims to provide guidelines for the use of multilayer algorithms of community detection on EEG-based brain multilayer networks. For this purpose, we tested and compared them on an artificial dataset that spans a wide range of network features.

We obtained our dataset by defining and implementing a tool able to generate pseudo-random multilayer networks with community structure. Among all the definitions of communities, we are considering the assortative one, namely, communities made of groups of nodes densely connected with each other and poorly connected with the other nodes of the network. In fact, previous findings have shown that this is a very plausible way with which nodes organize themselves in brain networks (Bertolero et al., 2015; Sporns and Betzel, 2016). With respect to the tools previously available in the literature (Lin et al., 2008; Kim and Han, 2009; Granell et al., 2015), we conceived this generator so that it can take as input as many settable parameters as possible; thus, we could be able to systematically test the algorithm under a variety of conditions and to evaluate the dependence of the performances on different factors. Specifically, a potential user can set as input the number of nodes, graph density, number of communities, ratio between intra-cluster and inter-cluster density, level of noise of the network, percentage of nodes shifting community across layers, and if the number of clusters diminishes, increases, or remains unchanged across layers. Thus, the main advantage of this generator is its flexibility in creating networks with different properties.

To test the algorithms, we simulated multilayer networks with features that are observable in brain functional networks estimated from EEG signals. We then considered two scenarios, one in which the community structure is stationary, the other is when it shows an evolution across the layers. While previous studies essentially focused on the second case, both cases are of interest in the neuroscience field.

In the first scenario, we aim to extract homogeneous community partitions among a certain number of noisy layers, and this could be useful when layers model either snapshots of a task in which the brain connectivity pattern is supposed to be stationary (with the only variations due to the noise) or groups of subjects with the same features. In this case, we seek for algorithms able to keep as stable as possible despite the presence of noise, one that, in an EEG-based network, could arise because of the variability of the signals, of the low SNR, or of the error intrinsic in any connectivity estimation procedure.

In the second scenario, we want our algorithm to track small and large variations in an evolving community structure. Examples of this scenario include when we want to discover the evolution of the modular organization underpinning cognitive functions causing time-varying connectivity patterns or relative to heterogeneous groups of people (e.g., healthy subjects and clinical cohorts). Here the capability of the methods to track the network's dynamics is the main feature we seek for.

The results of our extensive simulation studies show that all the algorithms are sensitive to the network features that we simulated. As expected, their performances decrease as the level of noise simulated increases because the community structure gets less and less clear. Moreover, their ability to exactly recover the imposed community structure diminishes when such structure is made of few clusters. This could happen because all the algorithms were introduced in a context other than neuroscience, where networks present thousands of nodes and many more clusters. In the case of time-varying communities, our analysis suggests that the proportion with which the clusters reconfigure does not affect consistently the algorithms' performances, except in a few cases in which, intuitively, the more it changes, the easiest the algorithms detect the variation. The genLouvain and, partially, the FacetNet algorithms were shown to be able to compensate for the presence of noise as the number of layers increases, returning more and more stable and accurate partitions in both scenarios explored here. Overall, genLouvain, which is based on multilayer modularity optimization, outperforms the others in most conditions. It has the best performances in most conditions. A single-layer modularity approach is also appropriate in case of few layers and low percentage of noise. FacetNet shows intermediate performances, as it seems to be able to mitigate the effect of a high level of noise when it has a high number of layers to work with.

Our work is not the first one attempting to address the issue of multilayer clustering algorithms' performances. In Silva et al. (2016) and Schmidt et al. (2018), the authors propose analysis with the same purpose. However, in the former, the focus is only on algorithms based on evolutionary clustering, which have been tested in a simple synthetic network and in three real networks not related to neuroscience. In the latter, the authors tested two approaches based on consensus clustering on a synthetic network. Such testing still has no statistical validity, as the two approaches have only been tested in one network, even if more realistic and closer to those experimentally estimated from EEG signals. Moreover, their main purpose was to exploit multilayer clustering approaches to threshold fully connected networks. For this reason, they introduced two new community detection algorithms, rather than considering the well-established multilayer optimization of modularity, which has already been proven to provide interesting insights in brain functioning and organization, as in Bassett et al. (2011). Another testing of the clustering algorithms has been done in Bazzi et al. (2020) on benchmark networks similar to those proposed here. However, the main focus of that work was on introducing a generative model for multilayer networks; therefore, the algorithms' performances were evaluated by only varying the coupling across layers. Here we performed a more comprehensive analysis: starting from preliminary analysis made to properly use the algorithms in different conditions determined by the network's properties, we compared the algorithms' behavior by systematically varying a set of the network's features, like cluster number, level of noise, coupling across layers, number of layers and network's density.

After having tested the algorithms on artificial networks, we applied them to a time-varying network obtained from a real EEG dataset under controlled conditions, from which we estimated multilayer networks, including a transition from one condition to the other. The experimental design has precise features designed to obtain accurate multilayer brain networks reflecting those simulated in the methodological analysis. Data was acquired from an adult healthy subject during a simple task, the resting state, composed by two distinct phases: OE and CE. The choice of taking a healthy subject rather than a patient spared us from making hard hypotheses on the underlying brain network. The same applies for the choice of the resting state, instead of more complicated cognitive or motor tasks, which would have required further hypotheses. At the same time, the two distinct consecutive phases (OE–CE) of the resting state guarantee a change in brain activity and, consequently, in brain connectivity and brain network, which is what we analyzed in the simulation study. Moreover, we established the number of EEG channels, the length of the trials, as well as their numerosity prior to the acquisition in order to obtain networks with the exact number of nodes and layers used in the simulation study. The data so collected have specific peculiarities that make it suitable for the validation of the algorithms' analysis. By applying the four algorithms on the obtained EEG multilayer networks, we could evaluate if, and how fast, such algorithms were able to recognize the two distinct phases. The results are consistent with what were found in simulations. GenLouvain outperforms the other algorithms by detecting stable communities within each condition and differences in the partitioning between the two conditions in the neighborhood of the transition. The topological representation of the community organization underlying the two conditions, shown in Figure 6, indicates that the closed eyes condition gives rise to a cluster of occipital electrodes which, during the open eyes condition, splits into two clusters, one for each hemisphere, and generally all the clusters become more hemisphere specific. This result is physiologically plausible. In fact, during the resting state at closed eyes, there is an increase of alpha rhythm associated with circuits originated in the occipital region, which disappear if the subjects open their eyes.

The purpose of the application to an EEG dataset was three-fold. First, it confirms the results obtained with the simulation studies. Moreover, as an indirect consequence, it validates the goodness of our model and of the generator with which we tested the algorithms, paving the way to its use in other studies. Finally, it supports the applicability of multilayer community detection to EEG-based brain networks. In fact, while several studies already showed the potentiality of employing graph theory instruments in EEG-derived networks to investigate brain functioning (Micheloyannis et al., 2006; Fallani et al., 2010; Toppi et al., 2012; Petti et al., 2016; Pichiorri et al., 2018), community detection and multilayer tools have been scarcely used in the electrophysiological context so far, despite promising results like those reported in a recent work (Kabbara et al., 2021) where authors investigated the modular structure of multilayer resting state networks with single-layer tools. Most of the studies on brain communities (e.g., Bassett et al., 2011; Betzel et al., 2014, 2017; Wig, 2017; Puxeddu et al., 2020) are conducted on brain networks obtained from functional magnetic resonance images (fMRI). fMRI data have the privilege of having a good spatial resolution. However, fMRI networks make a coarse assumption of stationarity. In fact, the BOLD signal peaks seconds after the neuronal activity, violating most of the brain information processing timescale, which ranges 100 ms (Park and Friston, 2013). EEG signals instead have a great temporal resolution, which is suited to the study of time-varying phenomena through a multilayer topological analysis. One could also think of invasive methods to obtain signals that are both spatially and temporally accurate. However, the invasiveness provides a strong limitation to the applicability of such an approach. Moreover, it currently allows to acquire data from a limited portion of the brain, failing to provide large-scale networks suited for an analysis of the communities which sustain the structure of most human brain functions. For these reasons, EEG-based brain networks represent a fair compromise between spatial and temporal resolution, and the study of their community structure can provide important insights into the brain dynamical organization.

After having studied the best practices and verified community detection applicability to multilayer EEG networks in a controlled case, future efforts will be put on studying how community structure evolves during tasks that elicit a dynamic configuration of the brain network. For this purpose, richer open EEG datasets could be investigated (i.e., van der Meer et al., 2016; Wong et al., 2018; Artoni et al., 2019), focused on resting state as well as more complex tasks, like working memory or auditory attention. This might provide physiological insights into brain functional organizational principles underlying cognitive functions.

Future investigations might also include the use of the toolbox that we provided to extend our analysis to other cases. For example, similar analysis could be performed in generating networks with a higher number of nodes and a higher number of clusters. Ultimately, this work could be useful in a cross-disciplinary way, regardless of our specific attention to EEG-based brain networks. The guidelines that we provide can be applied to every network with the simulated features, where community structure is supposed to be assortative.

## Conclusions

In conclusion, this work operated an extensive and systematic comparative analysis among multilayer community detection algorithms. We selected three different clustering approaches and four algorithms based on single-layer modularity, multilayer modularity, and evolutionary clustering. We tested them on artificial networks with modules generated through a toolbox defined for this purpose, which allows us to set most of the parameters characterizing the graphs that we systematically varied in a range typical of EEG-based brain networks to provide a comprehensive analysis of the algorithms. Specifically, we tested the algorithms' ability to recover stable and dynamic partitions out of multilayer networks with stationary and evolving community structure, respectively. Our results suggest that the performance of the algorithms depends on the network features, such as number of clusters, number of layers, and level of noise in the network. From the simulations, the community detection algorithm based on the optimization of the multilayer formulation of modularity turned out to be the most suitable within the explored conditions. The application of the algorithms to real networks estimated from EEG signals confirms these results and proves the applicability of such algorithms to electrophysiological data.

## Data Availability Statement

The raw data supporting the conclusions of this article will be made available by the authors, without undue reservation.

## Ethics Statement

The studies involving human participants were reviewed and approved by PROT. CE/PROG.685 on 13/06/2018. The participants provided their written informed consent to participate in this study.

## Author Contributions

MGP designed the study, implemented the codes, run the analysis, and wrote the original draft. MP designed the study and supervised the work. LA designed the study, supervised the work and provided funding. All authors revised and edited the manuscript.

## Conflict of Interest

The authors declare that the research was conducted in the absence of any commercial or financial relationships that could be construed as a potential conflict of interest.
