# Supplementary material for: A Comprehensive Analysis of Multilayer Community Detection Algorithms for Application to EEG-Based Brain Networks
Source: Front Syst Neurosci. 2021 Mar 1;15:624183. doi: 10.3389/fnsys.2021.624183 (PMC7956967; doi:10.3389/fnsys.2021.624183)
Supplement: Supplementary file 1 [file Data_Sheet_1.docx]

# SUPPLEMENTARY MATERIAL

# Content list

1. Preliminary investigation on the temporal resolution parameter in the multilayer modularity optimization algorithm, genLouvain (*[pag. 2](#_Preliminary_investigation_on)*)
2. Preliminary investigation on the trade-off parameter in multi-objective function optimization algorithm, FacetNet (*[pag. 8](#_Preliminary_investigation_on_1)*[)](#_Preliminary_investigation_on_1)
3. Simulation study: results unpacked (*[pag. 12](#_Simulation_study:_results)*)
4. Comparative analysis on networks with lower graph density (D=0.1) (*[pag. 16](#_Comparative_analysis_on)*)
5. Comparative analysis on networks with evolving community structure and with increasing or decreasing clusters number (*[pag. 22](#_Comparative_analysis_on_1)*)

# Preliminary investigation on the temporal resolution parameter in the multilayer modularity optimization algorithm, genLouvain

The genLouvain algorithm depends on two resolution parameters, γ and ω, which impact the dimension of the recovered clusters and their coupling across the layers, respectively. Several studies have already pointed out how the choice of γ affects modularity-based algorithms, and different strategies have been developed to select proper values. This work focuses on the multilayer aspect of the modules’ detection, so we focused on the behavior of genLouvain under different ω-values. Thus, we set γ to its default value (=1) and we explored the effects of different ω, both when community structure is stationary over the layers and when it changes. We performed two simulation studies similar to those described in Methods 2.2.1 and 2.2.2, but replacing the factor *algorithm* with the new factor *ω*. Specifically, we performed a repeated measures ANOVA with the factors *ω*=[0.1, 0.2, 0.5, 1, 2, 5, 10], CN=[2, 4, 6] (*number of clusters*), no=[10%, 25%, 50%] (*noise level* ), nL=[2, 10, 50, 100] (*number of layers*), and, for the case in which community structure is not stationary, also pn=[10%, 30%, 50%, 70%, 100%] (*percentage of nodes changing allegiance to modules*). Part of these results have been shown in [40]. The results of the ANOVA tests are reported in Tables 1 and 2.

In figure S1 we reported the results obtained in the first scenario (stationary community structure) for the index NMI_acc_. The algorithm shows high values of accuracy in networks with low noise level, number of clusters ≥ 4, irrespective of the ω-value. Higher values of ω (ω>0.5) are more suitable when the noise increases, especially in networks including several layers. Exploiting genLouvain with ω>0.5 guarantees a high level of accuracy even with *n*=50%. The analysis of the algorithm’s stability (Figure S2) led to analogous insights.

In figures S3 and S4 we reported the results obtained in the second scenario (evolving community structure), for NMI_acc_ and Dyn_ind_, respectively. In this case, the number of layers plays a key role in the choice of ω. Indeed, overall our results suggest that with time-varying community structure low ω-values are more suitable (ω<1) and provide higher accuracy of the output partition. On the contrary, when the change separates two community structures that are stable over several layers (e.g. when we are observing networks underlying two different tasks, or several subjects belonging to two different clinical conditions) higher ω-values are more advisable, especially with noisy networks. Intuitively, the lower the ω, the more genLouvain acts as a single-layer modularity algorithm, un-forcing the coupling of nodes across layers. Therefore, the accuracy of genLouvain used with ω<0.5 neither depends on the number of layers, nor on the factor *p*, and is extremely sensitive to the noise. A similar reasoning can be done for the dynamic index (Figure S4). In fact, lower ω-values are preferable in networks including few layers, meaning they tend to rapidly recover from rapid changes. Higher ω-values instead are better when the change separate two stationary conditions.

The results of this preliminary analysis have been used to run genLouvain with the proper ω, according to the particular features of the multilayer networks, in the algorithms comparison presented in the main paper.

| 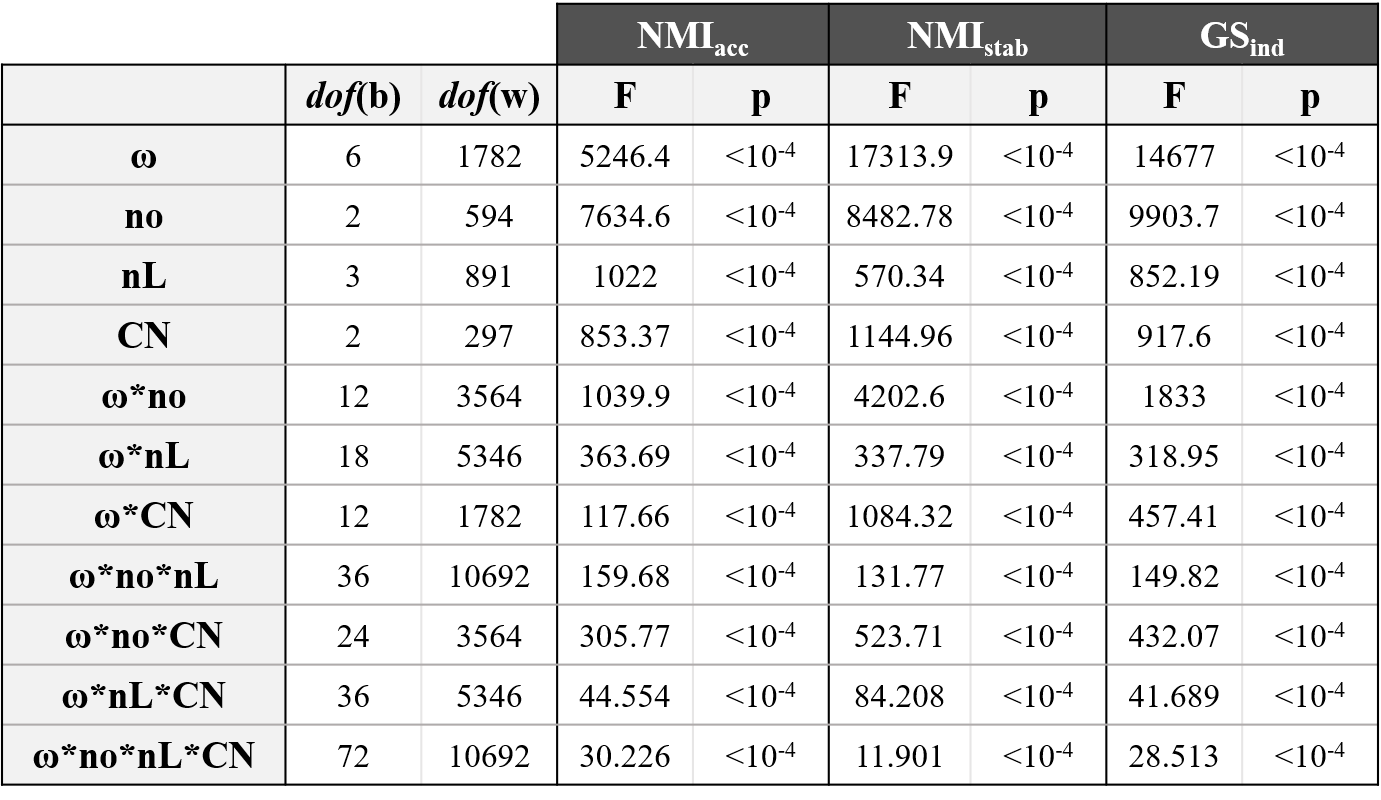 |
| --- |
| *Table S1. Results of the ANOVA test executed for the preliminary investigation on the temporal resolution parameter ω on networks with stationary community structure and graph density equal to 0.3. For each considered index (dependent variables of the test) we report the degrees of freedom (dof), F and p-values relative to single factors and the interactions among them.* |

| 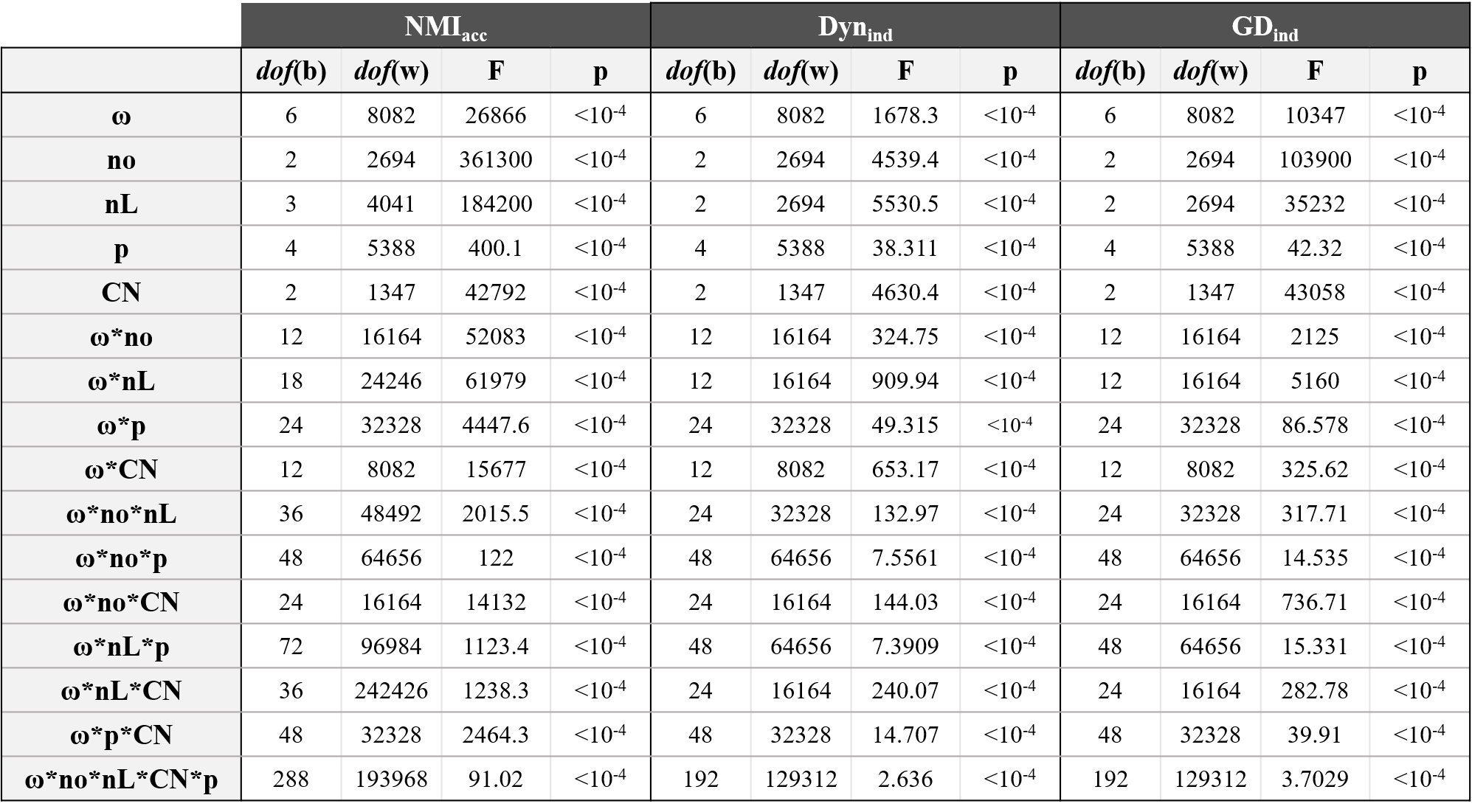 |
| --- |
| *Table S2. Results of the ANOVA test executed for the preliminary investigation on the temporal resolution parameter ω on networks with evolving community structure and graph density equal to 0.3. For each considered index (dependent variables of the test) we report the degrees of freedom (dof), F and p-values relative to single factors and the interactions among them.* |

| 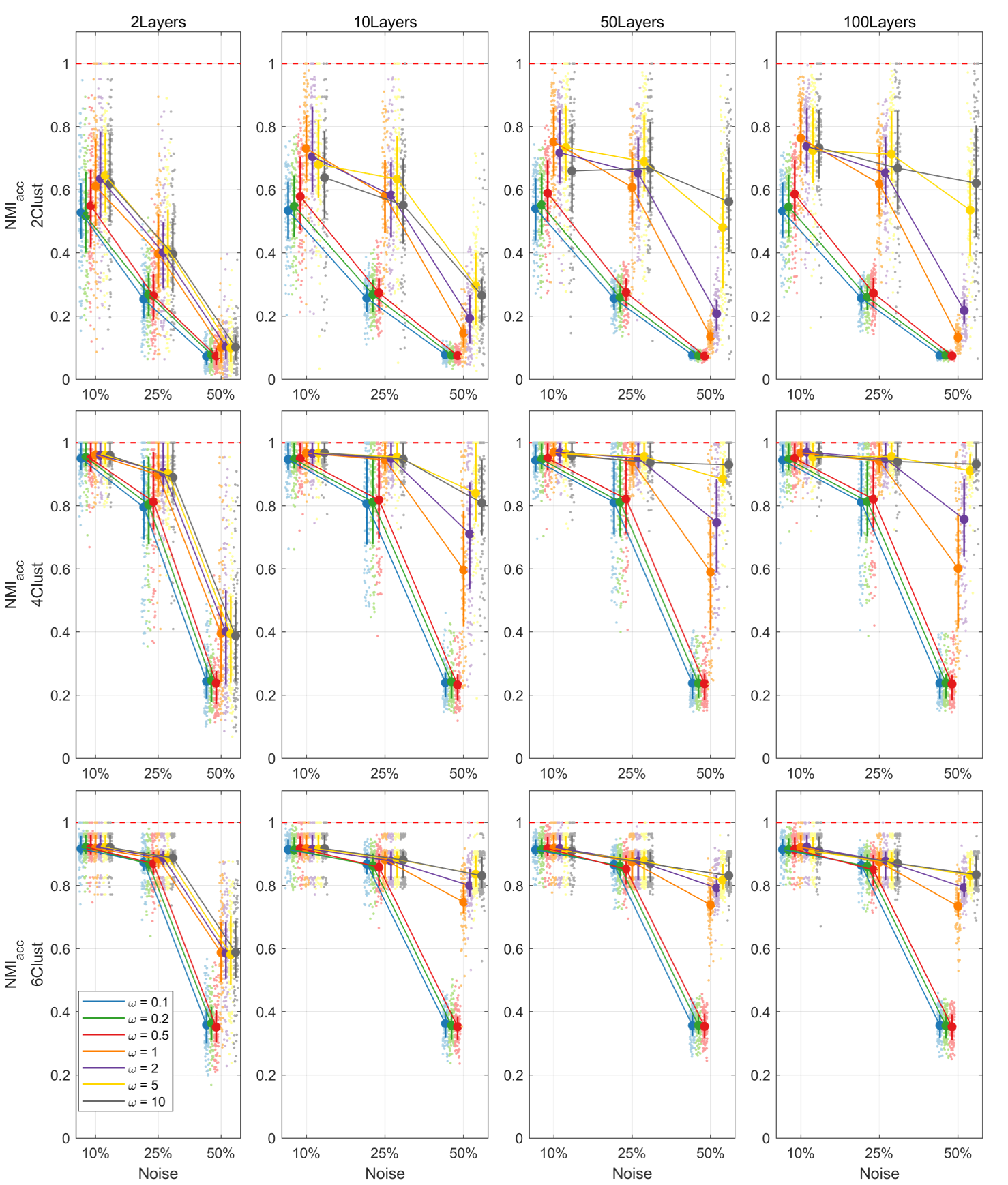 |
| --- |
| Figure S1. *Plot of means and standard deviations of* ${NMI}_{acc}$ *in the preliminary analysis regarding genlouvain on networks with stationary community structure. In the rows and the columns of the pictures we report the results for different levels of number of clusters (CN) and number of layers (nL) respectively. In each subplot the* ${NMI}_{acc}$ *of each algorithm, identified with colors code, is shown for the different levels of percentage of noise (no).* |

| 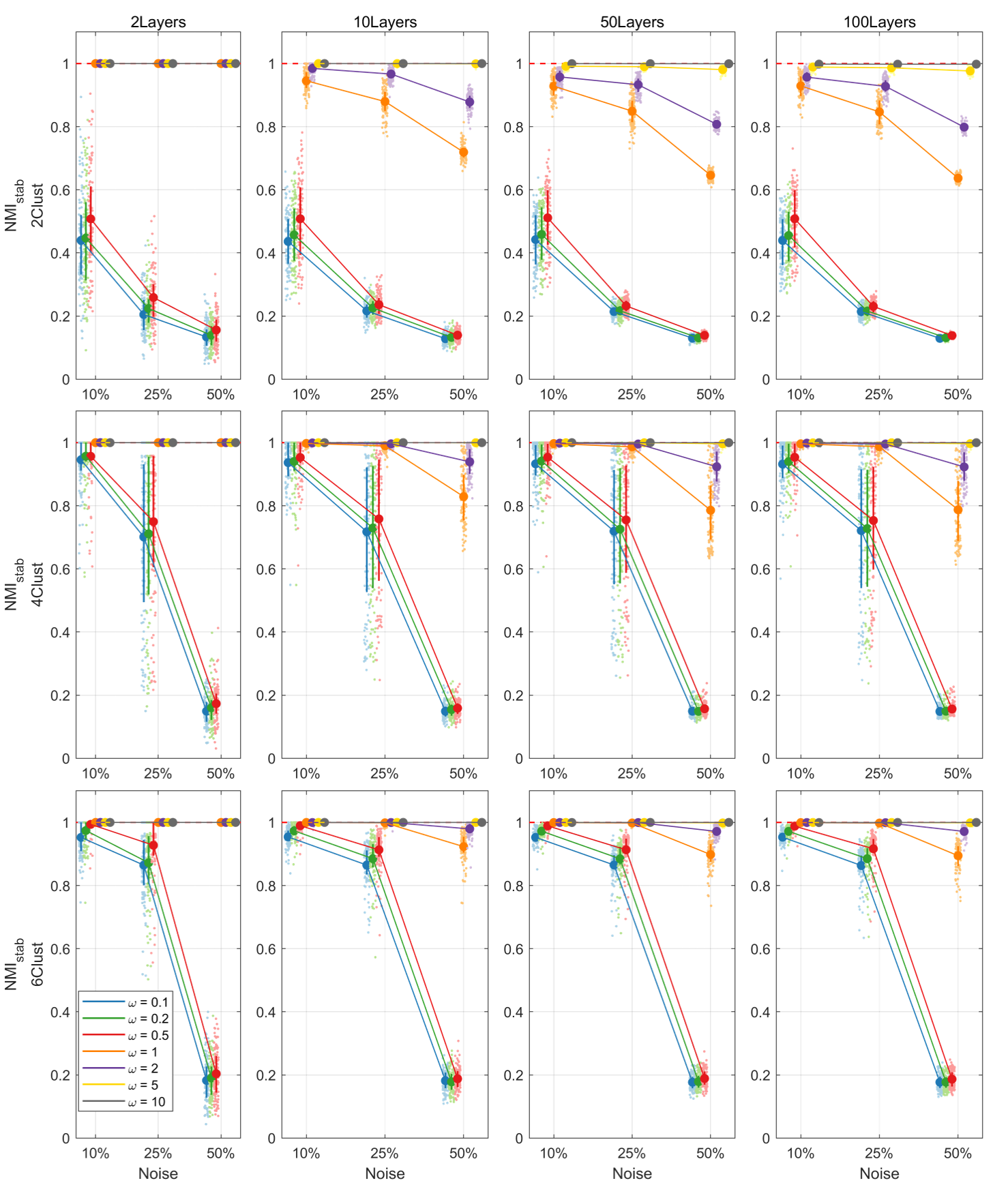 |
| --- |
| Figure S2. *Plot of means and standard deviations of* ${NMI}_{stab}$ *in the preliminary analysis regarding genlouvain on networks with stationary community structure. In the rows and the columns of the pictures we report the results for different levels of number of clusters (CN) and number of layers (nL) respectively. In each subplot the* ${NMI}_{stab}$ *of each algorithm, identified with colors code, is shown for the different levels of percentage of noise (no).* |

| 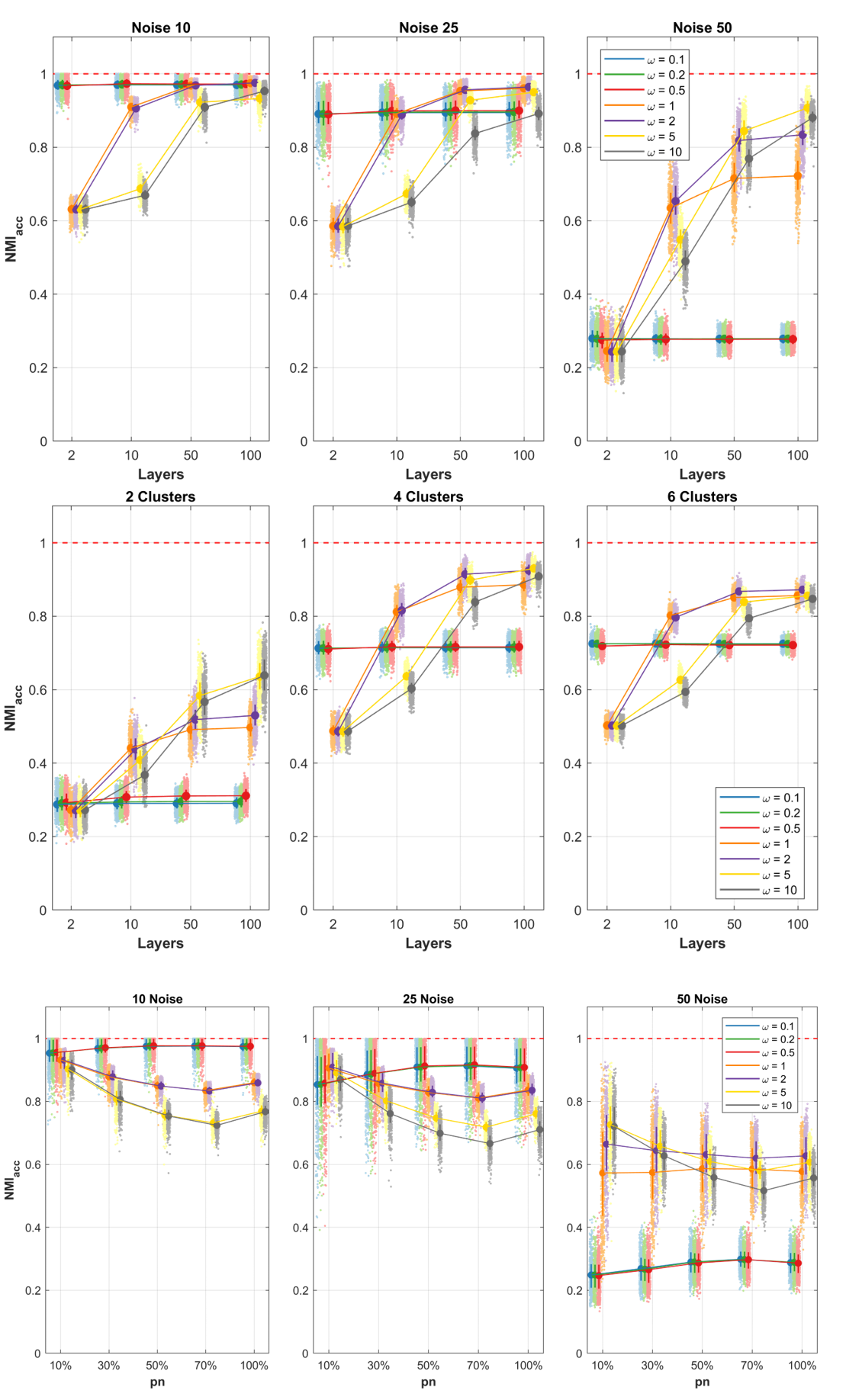 |
| --- |
| *Figure S3. Plot of means and standard deviations of* ${NMI}_{acc}$ *in the preliminary analysis regarding genlouvain on networks with evolving community structure. In the first row we report the accuracy of the algorithms, identified with different colors, with respect to the different levels of number of layers, x-axis, and percentage of noise, columns. In the second row we show the trend of the algorithms’ accuracies with respect to the number of layers, x-axis, and number of clusters, columns. In the third row we reported the accuracies mean values for each algorithm to varying of the factor pn, x-axis, and level of noise, columns.* |

| 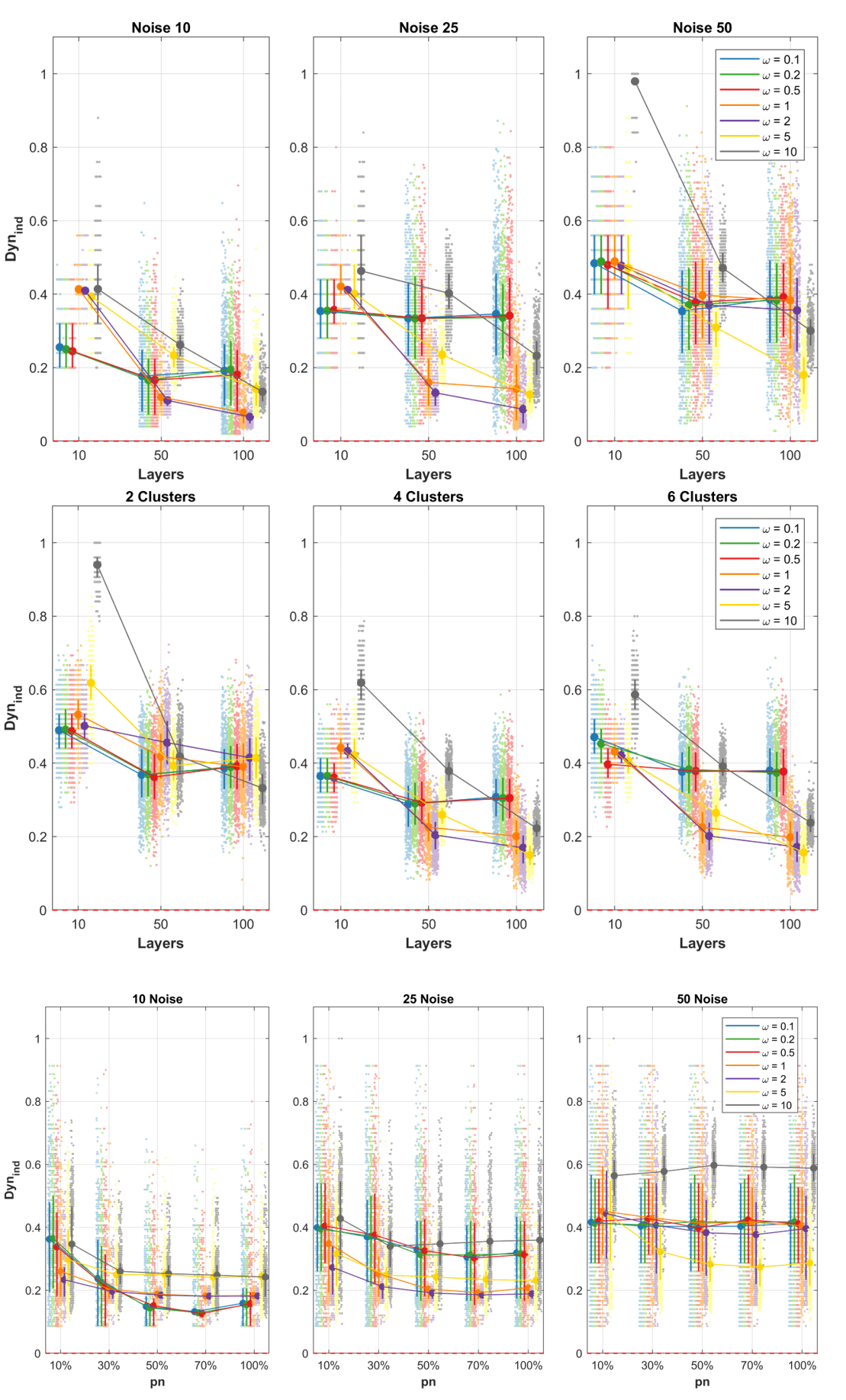 |
| --- |
| *Figure S4. Plot of means and standard deviations of* ${Dyn}_{ind}$ *in the preliminary analysis regarding genlouvain on networks with evolving community structure. In the first row we report the dynamic of the algorithms, identified with different colors, with respect to the different levels of number of layers, x-axis, and percentage of noise, columns. In the second row we show the trend of the algorithms’ speed with respect to the number of layers, x-axis, and number of clusters, columns. In the third row we reported the* ${Dyn}_{ind}$ *mean values for each algorithm to varying of the factor pn, x-axis, and level of noise, columns.* |

# Preliminary investigation on the trade-off parameter in multi-objective function optimization algorithm, FacetNet

The FacetNet algorithm depends on a trade-off parameter λ that balances the accuracy of the partitions at each layer and their coupling over the layers. It can vary between 0 (no coupling among layers) and 1 (coupling across layers prevails over single-layer accuracy). We explored the behavior of FacetNet for different values of λ in the stationary and evolving community structure scenarios. As in the previous paragraph, we performed two simulation studies as in Methods 2.2.1 and 2.2.2, replacing the factor *algorithm* with the factor *λ*. In analogy with the ω investigation, we performed a repeated measures ANOVA with the factors λ = [0.1, 0.2, 0.5, 0.7, 0.8, 0.9, 1], CN=[2, 4, 6] (*number of clusters*), no=[10%, 25%, 50%] (*noise level*), nL=[2, 10, 50, 100] (*number of layers*), and for the case in which community structure is not stationary also pn=[10%, 30%, 50%, 70%, 100%] (*percentage of nodes changing allegiance to modules*). Results of the statistic tests are reported in Tables 3 and 4.

We show the results in the case of stationary community structure in Figures S5 and S6, where we show the algorithm’s accuracy and stability, respectively. In this case higher λ-values are always preferable. However, choosing λ=1, despite provides excellent stability of the algorithm’s output, results in lower accuracy. Thus, the most suitable values are 0.8 or 0.9. Regardless of the specific λ, the algorithm is sensitive to the noise level and the number of layers, being more accurate and stable when operating in multilayer networks with several layers and low noise level.

The results obtained on networks with evolving community structure are reported in Figure S7 and S8. As for Dyn_ind_, lower λ-values are always preferable. On the other hand, for the accuracy choosing higher λ-values (from 0.5 to 0.9) provides better outputs in noisy networks made of many layers. On the contrary, if the network is not noisy or it has few layers (e.g. from 2 to 10) lower values are more suitable. Globally, with every λ-value used, except λ=1, the algorithm is sensitive to the level of noise and the number of clusters, achieving better performance when it has to detect a number of clusters >2 in non-noisy networks. With intermediate λ-values in the range [0.5, 0.9], FacetNet’s accuracy is also proportional dependent on the number of layers.

The results of this preliminary analysis have been used to run FacetNet with the proper λ-value, according to the specific features of the multilayer networks, in the algorithms comparison presented in the main paper.

| 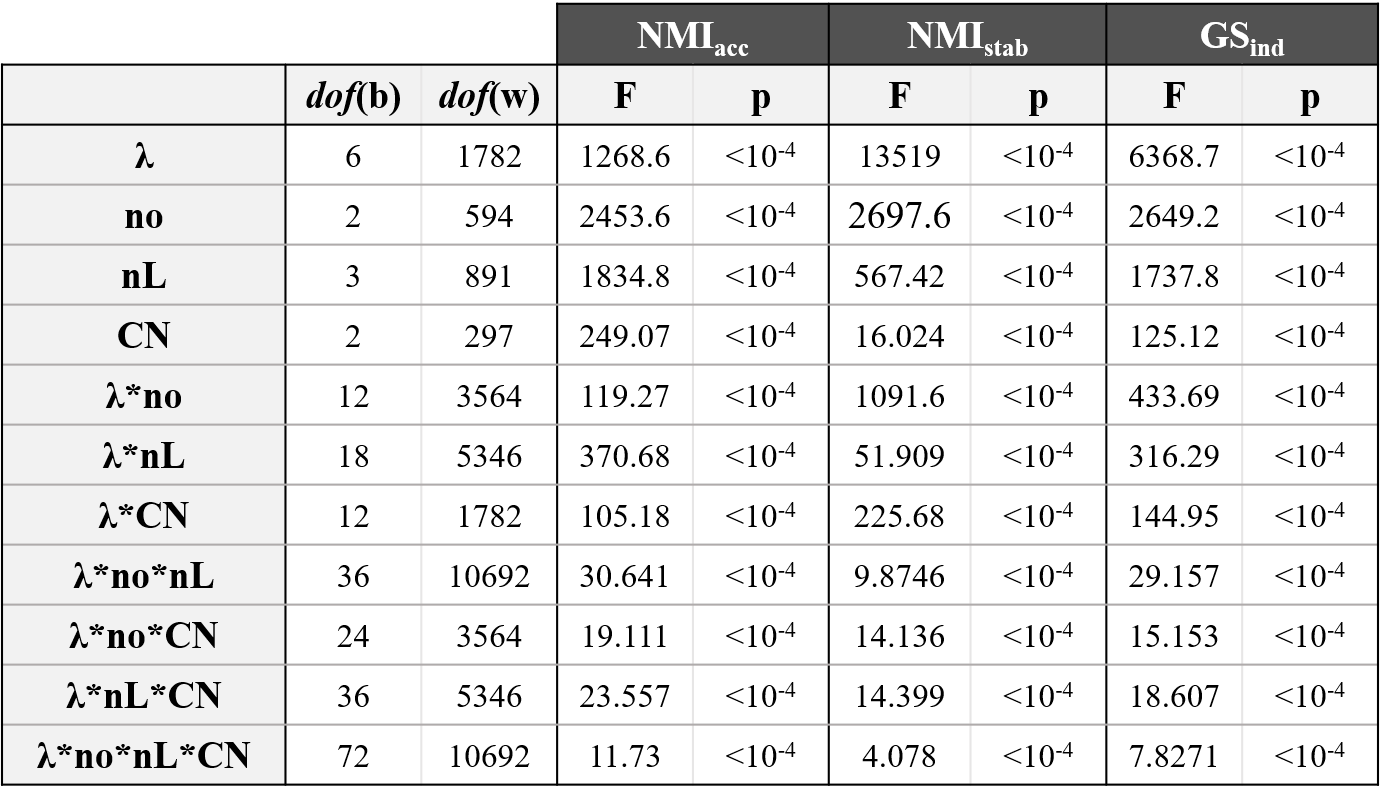 |
| --- |
| *Table S3. Results of the ANOVA test executed for the preliminary investigation on the trade-off parameter λ on networks with stationary community structure and graph density equal to 0.3. For each considered index (dependent variables of the test) we report the degrees of freedom (dof), F and p-values relative to single factors and the interactions among them.* |

| 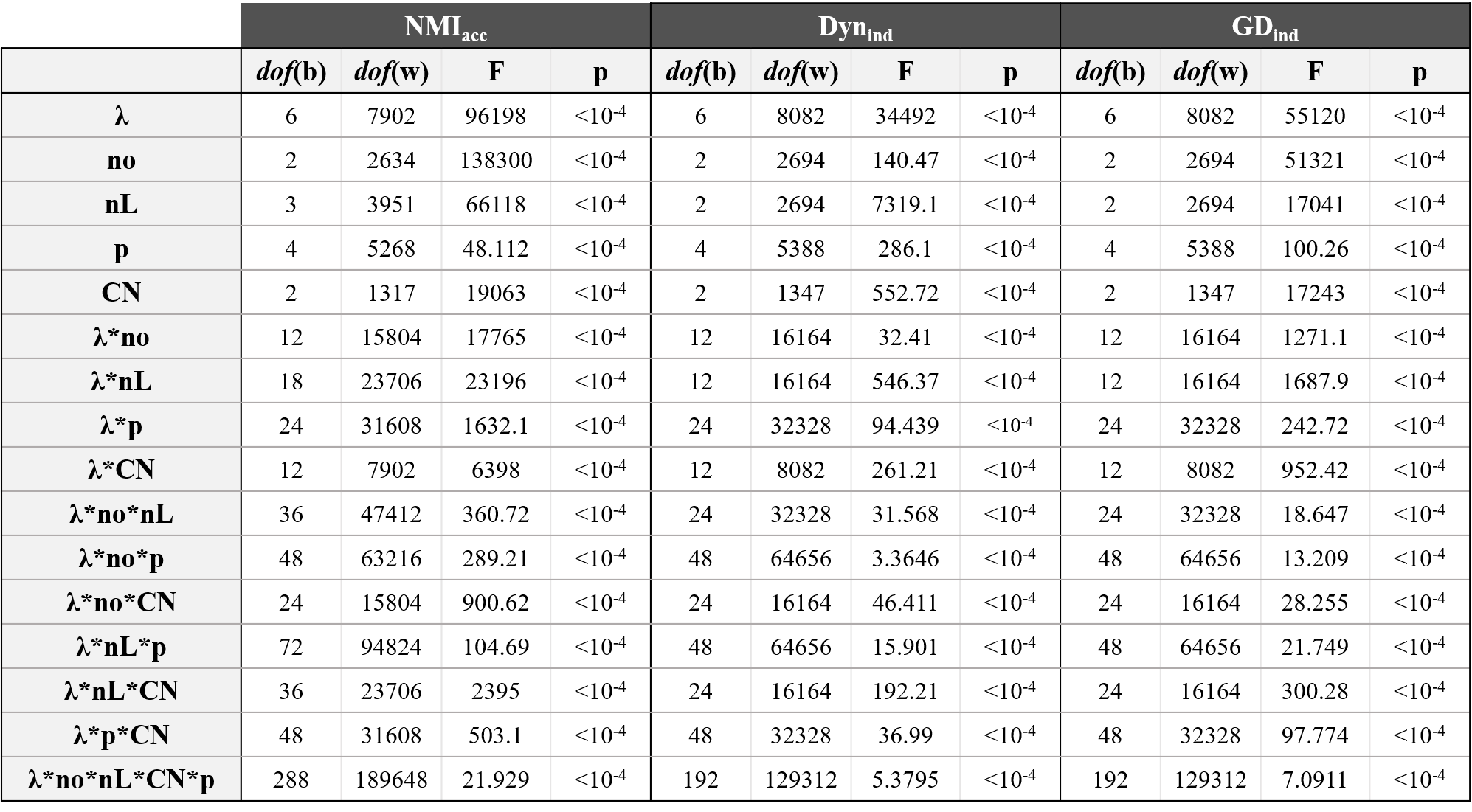 |
| --- |
| *Table S4. Results of the ANOVA test executed for the preliminary investigation on the trade-off parameter λ on networks with evolving community structure and graph density equal to 0.3. For each considered index (dependent variables of the test) we report the degrees of freedom (dof), F and p-values relative to single factors and the interactions among them.* |

| 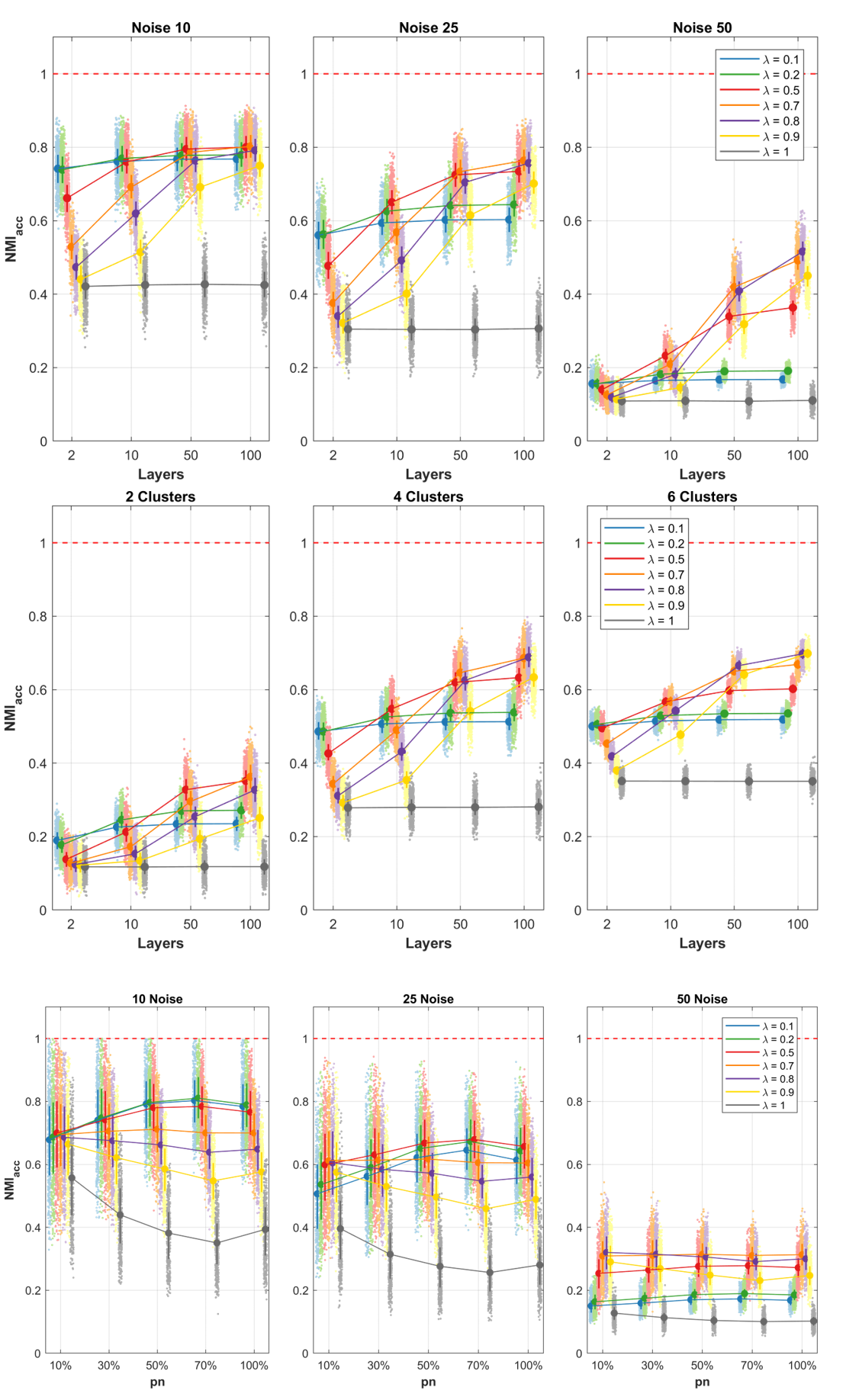 |
| --- |
| *Figure S7. Plot of means and standard deviations of* ${NMI}_{acc}$ *in the preliminary analysis regarding FacetNet on networks with evolving community structure. In the first row we report the accuracy of the algorithms, identified with different colors, with respect to the different levels of number of layers, x-axis, and percentage of noise, columns. In the second row we show the trend of the algorithms’ accuracies with respect to the number of layers, x-axis, and number of clusters, columns. In the third row we represent the accuracies mean values for each algorithm to varying of the factor pn, x-axis, and level of noise, columns.* |

| 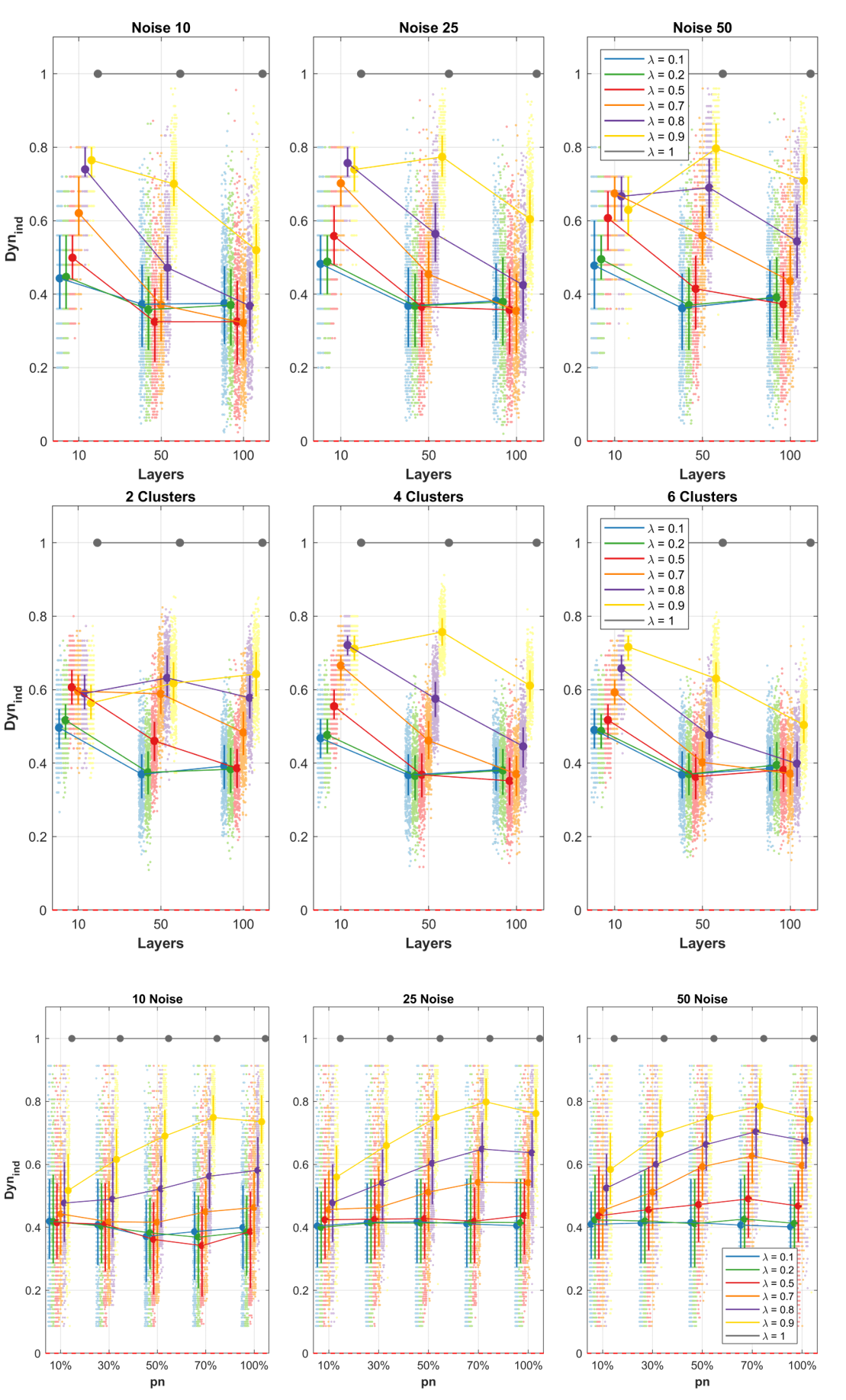 |
| --- |
| *Figure S8. Plot of means and standard deviations of* ${Dyn}_{ind}$ *in the preliminary analysis regarding FacetNet on networks with evolving community structure. In the first row we report the dynamic of the algorithms, identified with different colors, with respect to the different levels of number of layers, x-axis, and percentage of noise, columns. In the second row we show the trend of the algorithms’ speed with respect to the number of layers, x-axis, and number of clusters, columns. In the third row we represent the* ${Dyn}_{ind}$ *mean values for each algorithm to varying of the factor pn, x-axis, and level of noise, columns.* |

# Simulation study: results unpacked

| 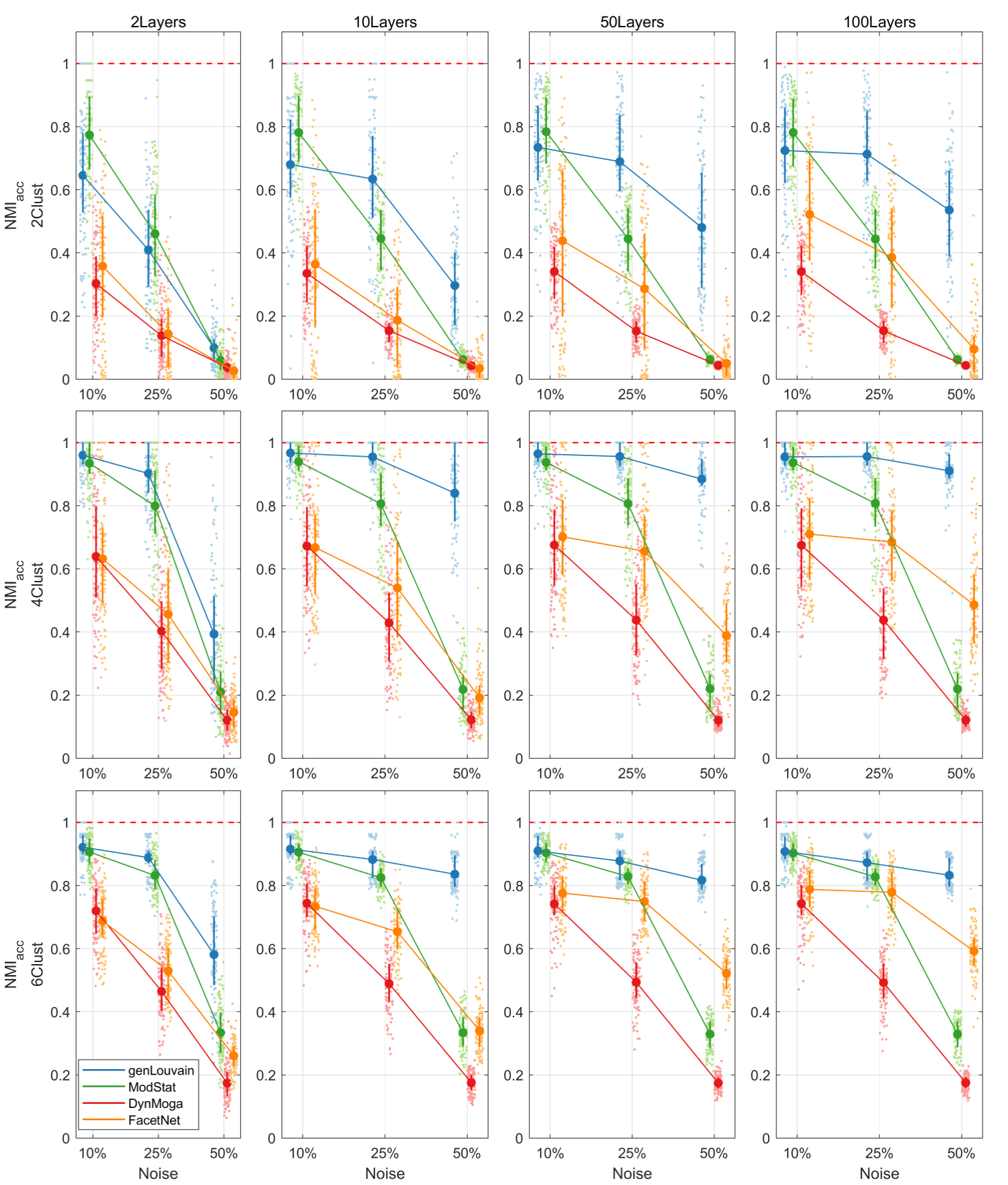 |
| --- |
| Figure S9. Plot of means and standard deviations of ${NMI}_{acc}$ in the comparative analysis on networks with stationary community structure. In the rows and the columns of the pictures we report the results for different levels of clusters number (CN) and number of layers (nL) respectively. In each subplot the ${NMI}_{acc}$ of each algorithm, identified with colors code, is shown for the different levels of percentage of noise (no). |

While in Figure 3-6 of the main manuscript we report the algorithms performances with respect to one ANOVA factor, irrespective of the others, here the same results are unpacked and we can observe algorithms performances for each combination of the three ANOVA factors.

| 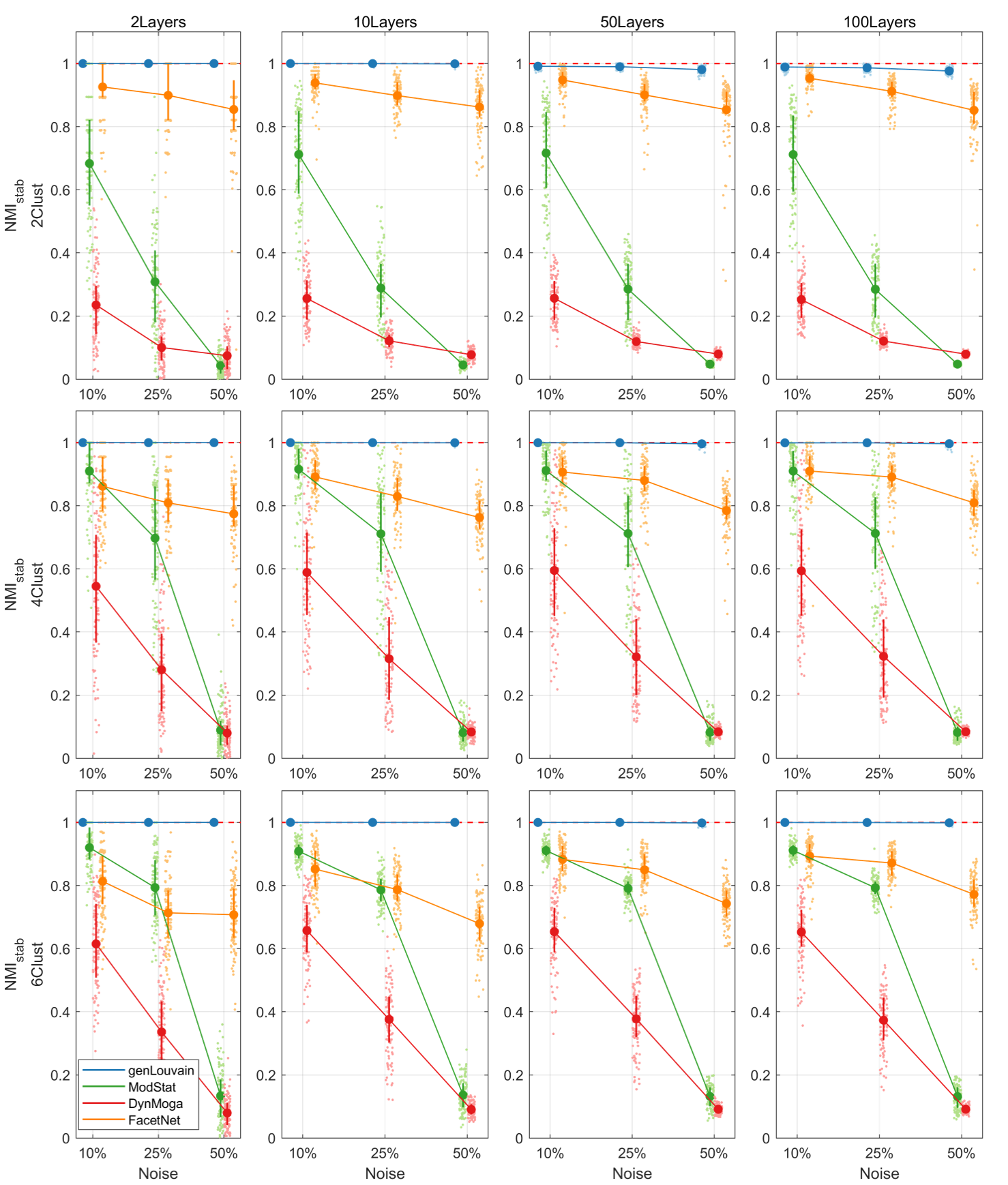 |
| --- |
| Figure S10. Plot of means and standard deviations of ${NMI}_{stab}$ in the comparative analysis on networks with stationary community structure. In the rows and the columns of the pictures we report the results for different levels of clusters number (CN) and number of layers (nL) respectively. In each subplot the ${NMI}_{stab}$ of each algorithm, identified with colors code, is shown for the different levels of percentage of noise (no). |

| 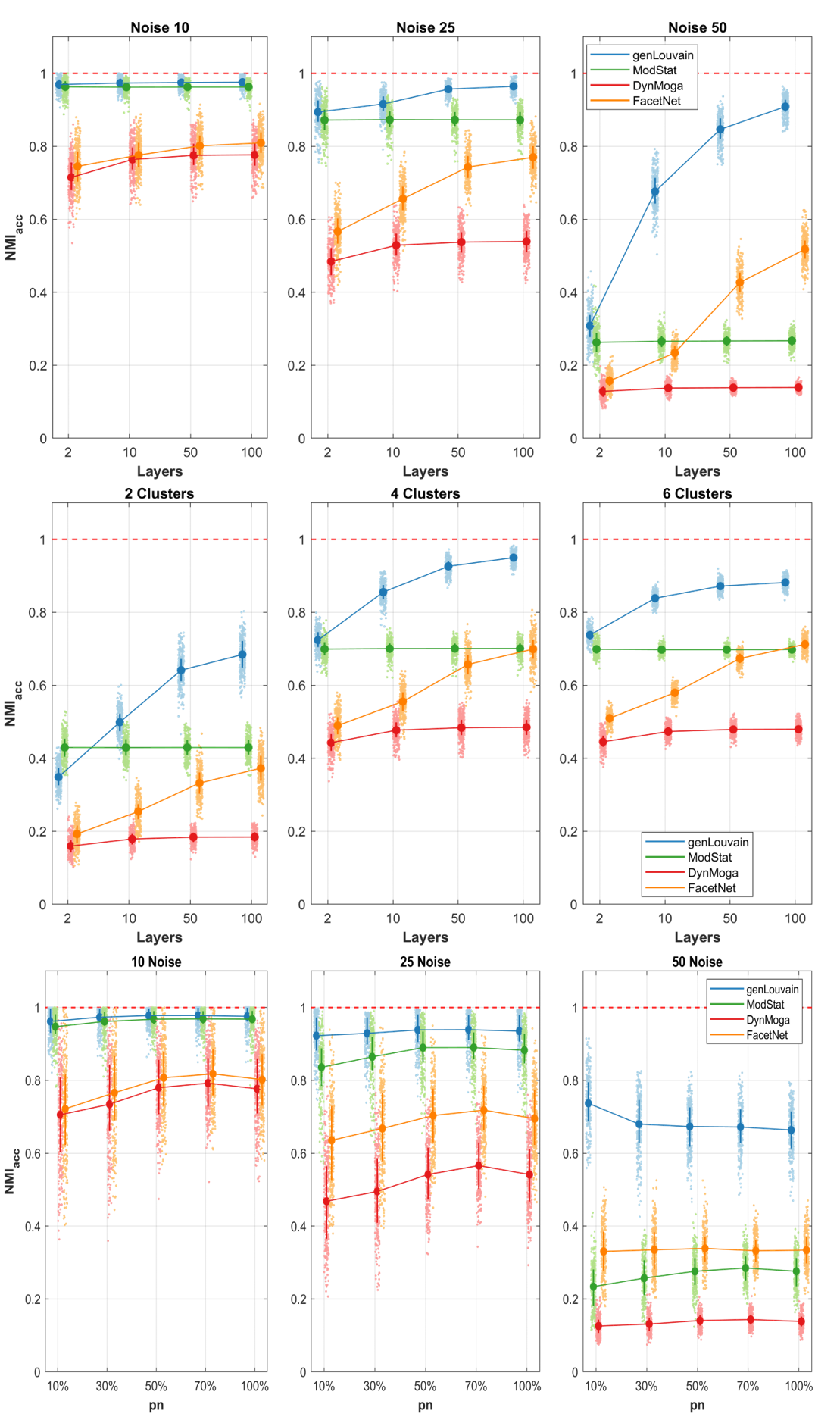 |
| --- |
| Figure S11. Plot of means and standard deviations of ${NMI}_{acc}$ in the comparative analysis on networks with evolving community structure. In the first row we report the accuracy of the algorithms, identified with different colors, with respect to the different levels of number of layers, x-axis, and percentage of noise, columns. In the second row we show the trend of the algorithms’ accuracies with respect to the number of layers, x-axis, and clusters number, columns. In the third row we represent the accuracies mean values for each algorithm to varying of the factor pn, x-axis, and level of noise, columns. |

| 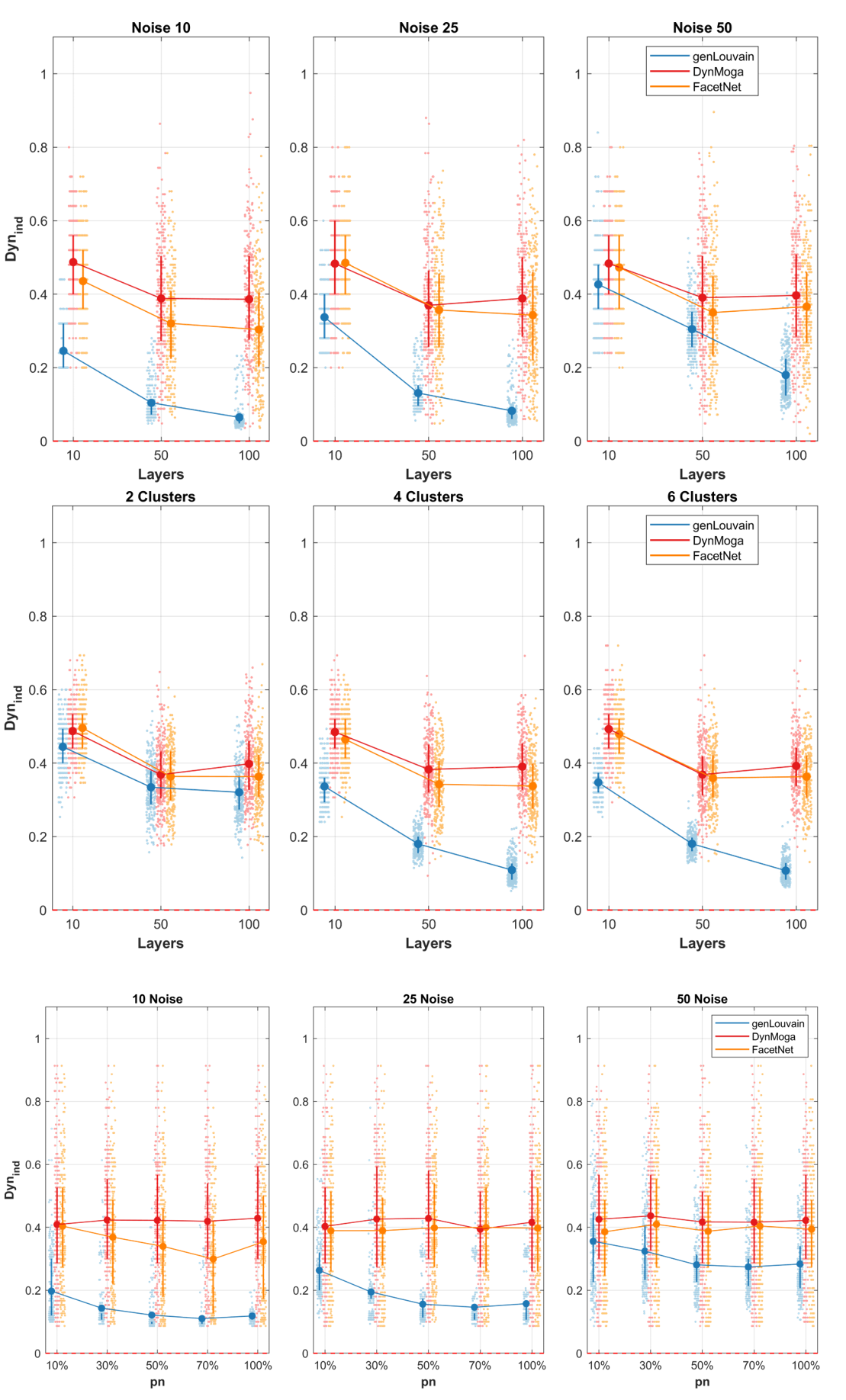 |
| --- |
| Figure S12. Plot of means and standard deviations of ${Dyn}_{ind}$ in the comparative analysis on networks with evolving community structure. In the first row we report the dynamic of the algorithms, identified with different colors, with respect to the different levels of number of layers, x-axis, and percentage of noise, columns. In the second row we show the trend of the algorithms’ speed with respect to the number of layers, x-axis, and clusters number, columns. In the third row we represent the ${Dyn}_{ind}$ mean values for each algorithm to varying of the factor pn, x-axis, and level of noise, columns. |

# Comparative analysis on networks with lower graph density (D=0.1)

In this section we present the results of the comparative simulation studies (Section 2 of the main paper) with a network density D=0.1. In figures S9 and S10 we reported the results of the study made on networks with stationary community structure, while in figures S11 and S12 those of the study made on networks with evolving community structure (as described in the Methods sections 2.2.1 and 2.2.2, respectively). In details, we generated networks with D=0.1 and we performed a repeated measures ANOVA with the factors *algorithm*={genLouvain, ModStat, DynMoga, FacetNet}, CN=[2, 4, 6] (*number of clusters*), no=[10%, 25%, 50%] (*noise level*), nL=[2, 10, 50, 100] (*number of layers*), and for the second case in which community structure is not stationary also pn=[10%, 30%, 50%, 70%, 100%] (*percentage of nodes changing allegiance to modules*). The results of these ANOVA tests are reported in Tables 5 and 6.

In both cases (stationary and evolving community structure) the trends of the algorithms’ accuracy and stability/dynamicity over the different combinations of the factors do not change substantially with respect to the analysis shown in the main paper for D=0.3 (Figures 3-6). GenLouvain is the most suitable algorithm for the detection of assortative communities (i.e. modules) in multilayer networks, in each simulated condition. However, ModSat equates genLouvain’s performance if the multilayer networks are composed by few layers (nL<10) and the community structure is not noisy (no=10%). Overall, all the algorithms show higher performance when operating on networks composed by an increasing number of clusters and decreasing level of noise. Trivially, the behavior of ModStat does not depend on the number of layers constituting the multilayer networks, since it operates by maximizing the modularity on every single independently. On the contrary, GenLouvain and FacetNet are sensitive to nL, to the extent in which their performance (in terms of accuracy and stability/dynamics) increase together with this variable.

The main difference with denser networks (D=0.3) is that with lower density the performances of all the algorithms are globally reduced, especially when the networks are made of few clusters (i.e. 2 clusters).

| 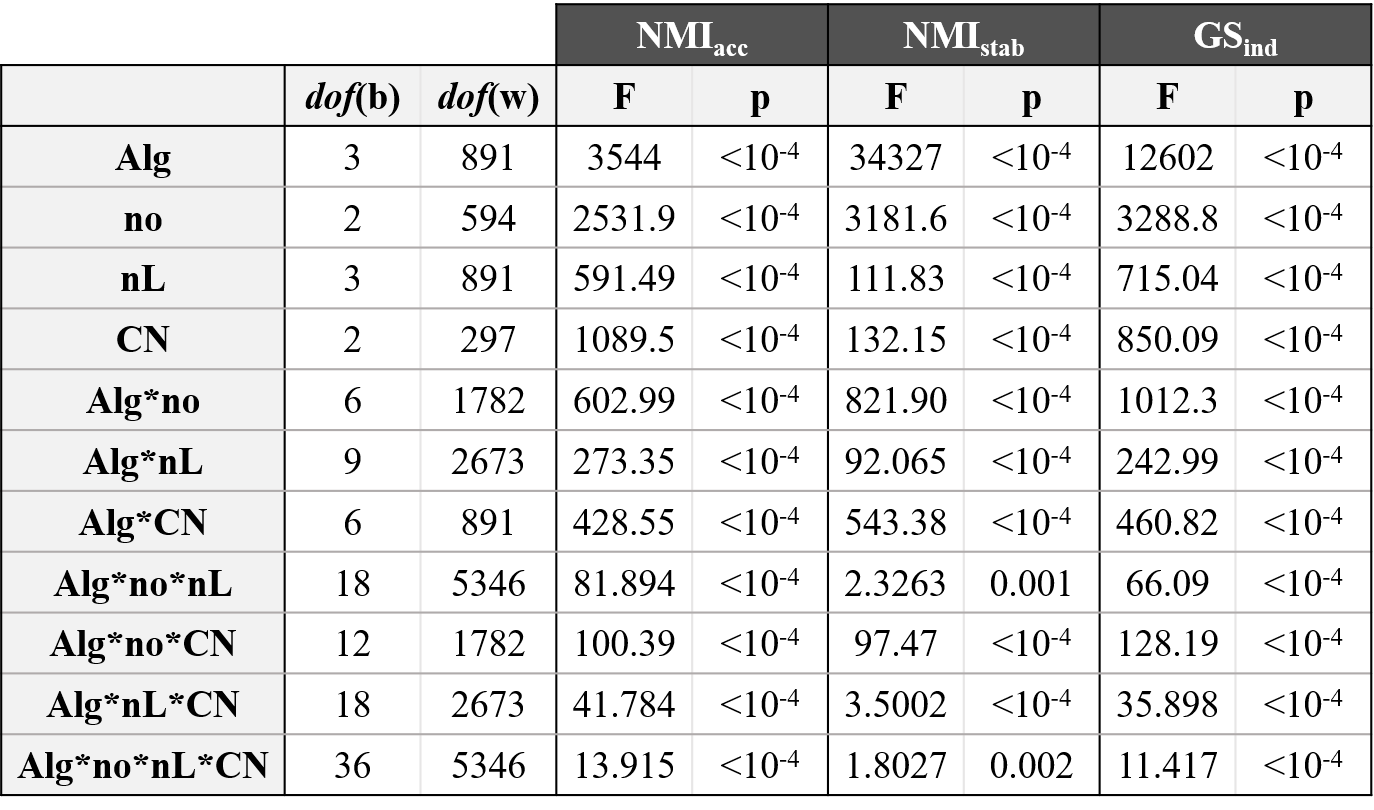 |
| --- |
| *Table S5. Results of the ANOVA test executed for the comparative analysis on networks with stationary community structure and graph density equal to 0.1. For each considered index (dependent variables of the test) we report the degrees of freedom (dof), F and p-values relative to single factors and the interactions among them.* |

| 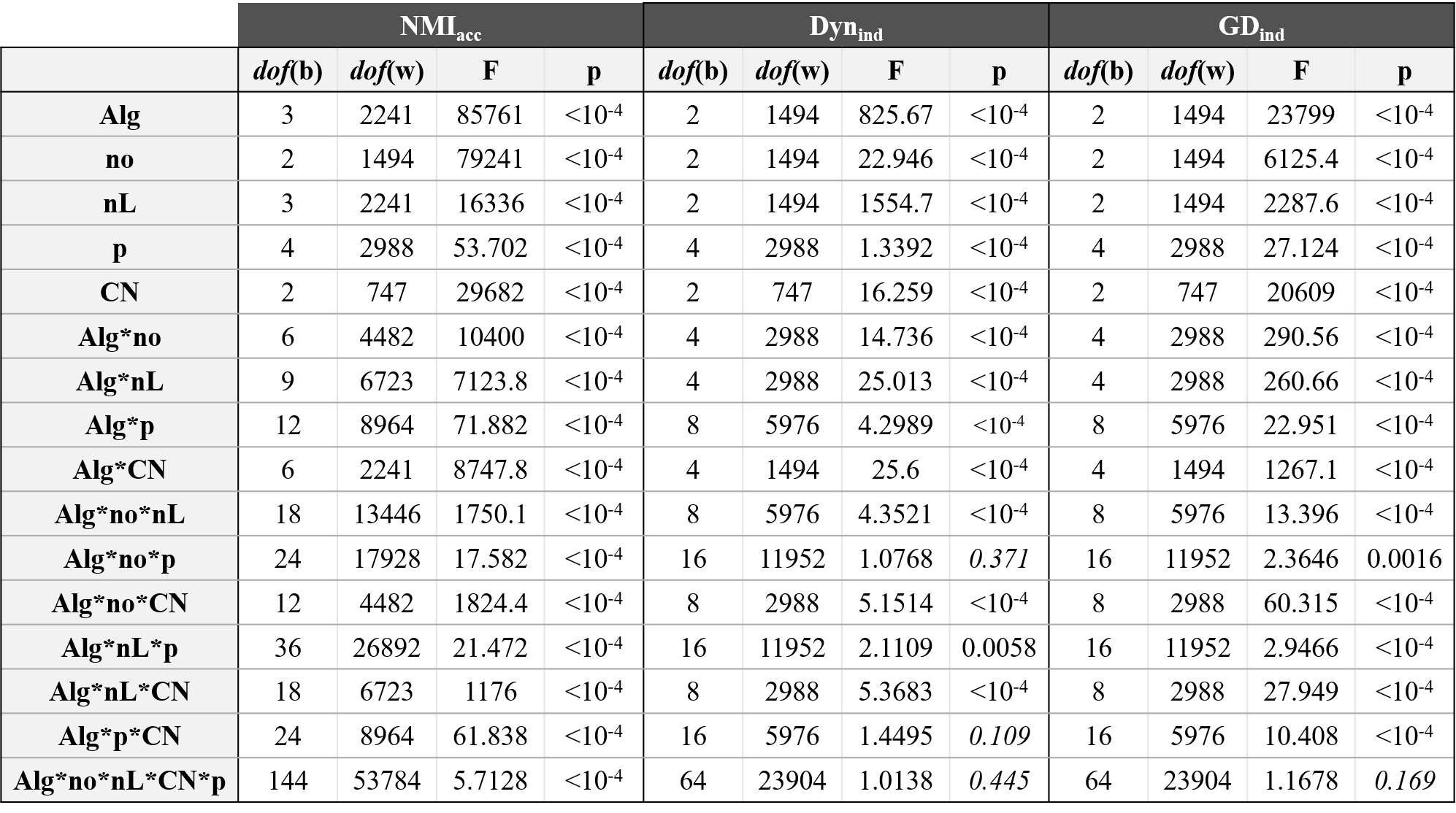 |
| --- |
| *Table S6. Results of the ANOVA test executed for the comparative analysis on networks with evolving community structure and graph density equal to 0.1. For each considered index (dependent variables of the test) we report the degrees of freedom (dof), F and p-values relative to single factors and the interactions among them* |

| 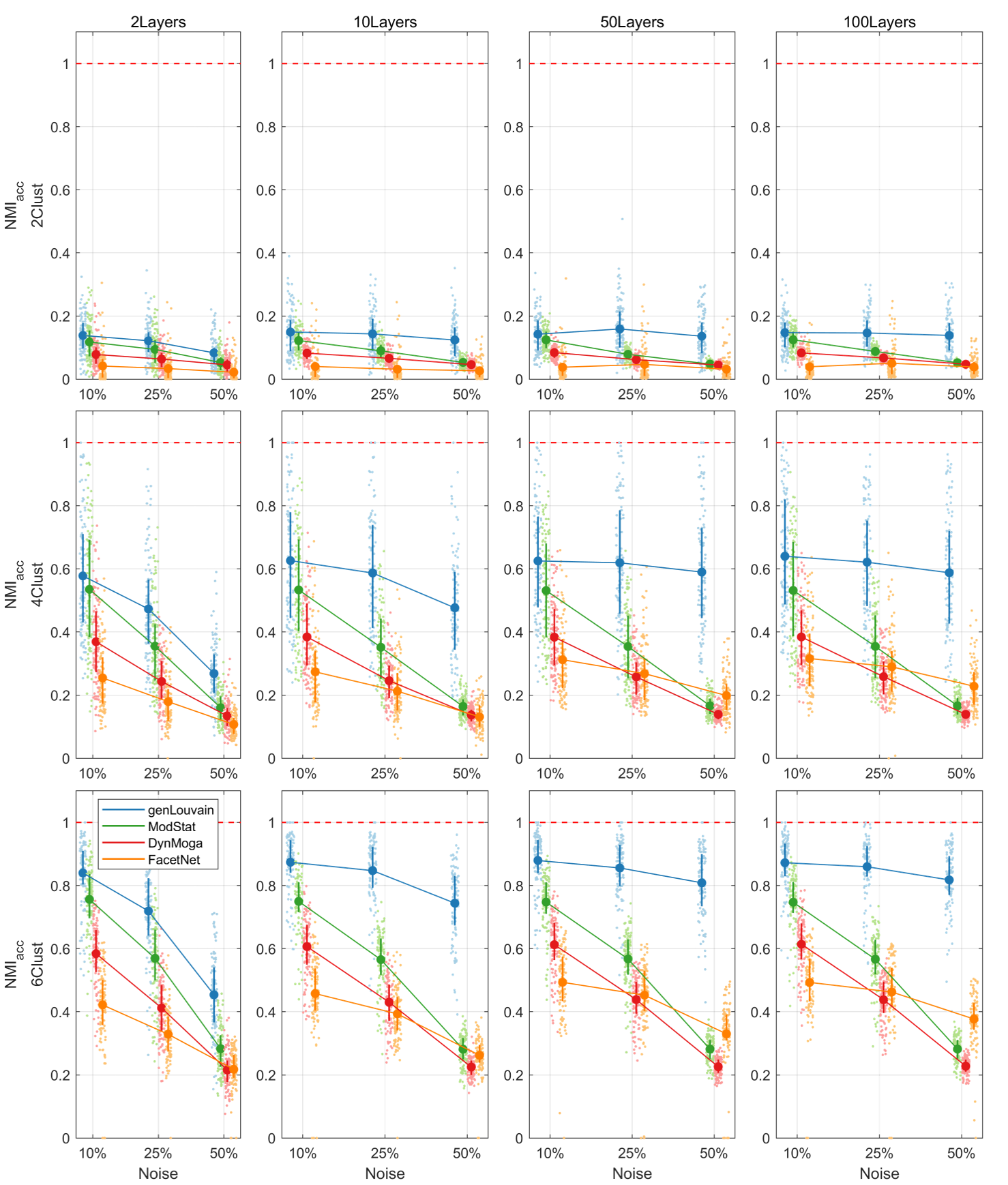 |
| --- |
| Figure S13. *Plot of means and standard deviations of* ${NMI}_{acc}$ *in the comparative analysis on networks with D=0.1 and stationary community structure. In the rows and the columns of the pictures we report the results for different levels of number of clusters (CN) and number of layers (nL) respectively. In each subplot the* ${NMI}_{acc}$ *of each algorithm, identified with colors code, is shown for the different levels of percentage of noise (no).* |

| 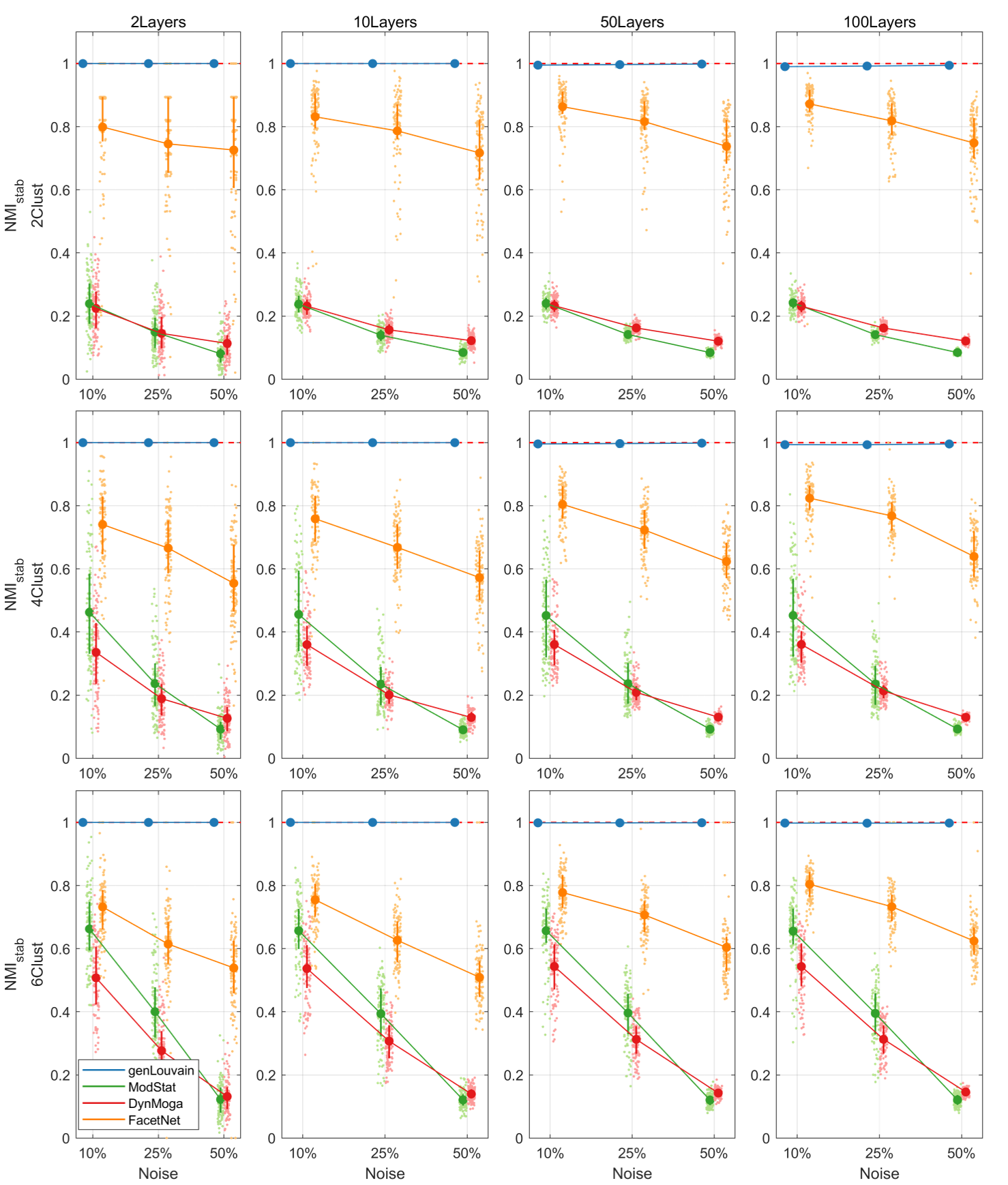 |
| --- |
| *Figure S14. Plot of means and standard deviations of* ${NMI}_{stab}$ *in the comparative analysis on networks with D=0.1 and stationary community structure. In the rows and the columns of the pictures we report the results for different levels of number of clusters (CN) and number of layers (nL) respectively. In each subplot the* ${NMI}_{stab}$ *of each algorithm, identified with colors code, is shown for the different levels of percentage of noise (no).* |

| 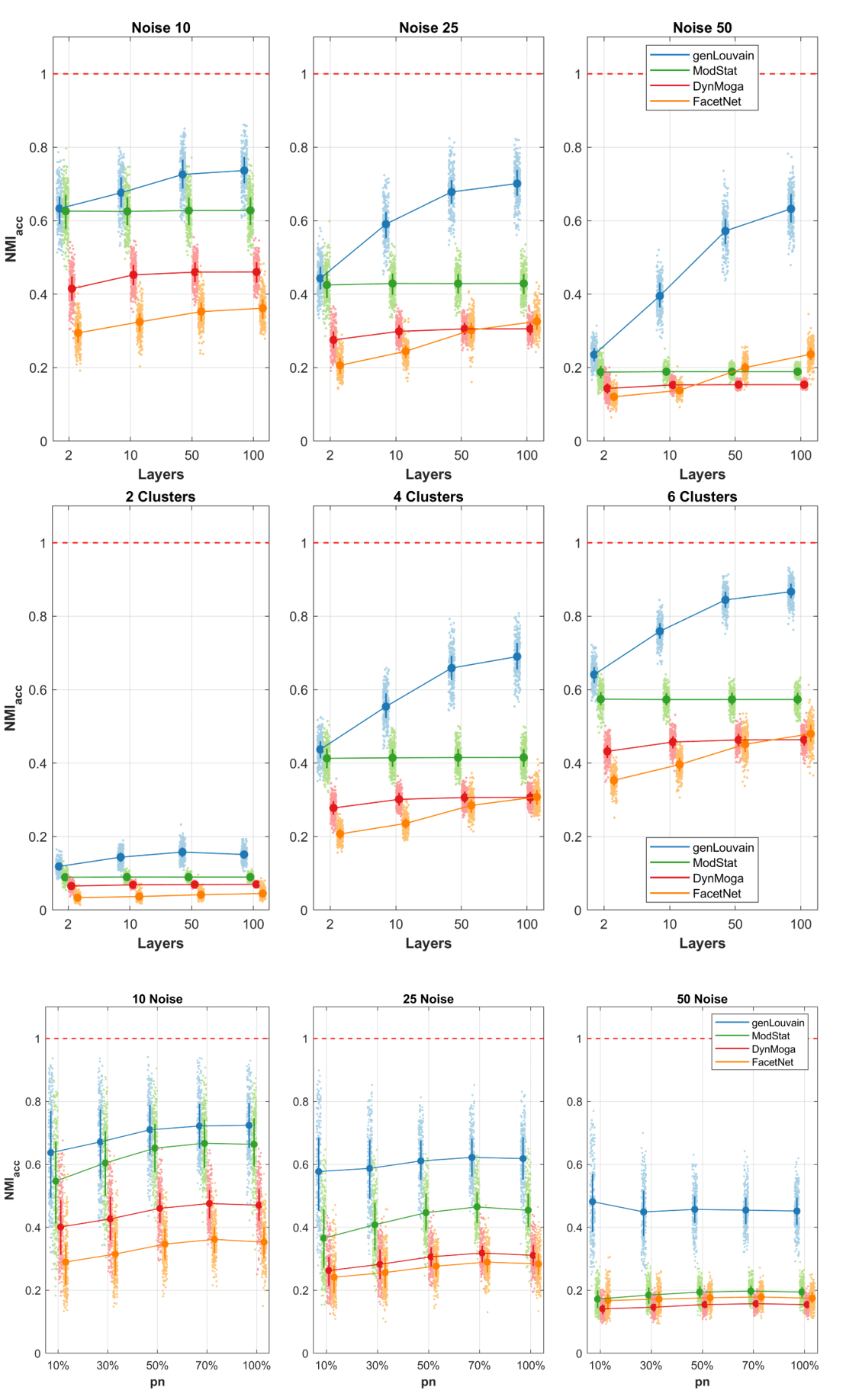 |
| --- |
| *Figure S15. Plot of means and standard deviations of* ${NMI}_{acc}$ *in the comparative analysis on networks with D=0.1 evolving community structure. In the first row we report the accuracy of the algorithms, identified with different colors, with respect to the different levels of number of layers, x-axis, and percentage of noise, columns. In the second row we show the trend of the algorithms’ accuracies with respect to the number of layers, x-axis, and number of clusters, columns. In the third row we represent the accuracies mean values for each algorithm to varying of the factor pn, x-axis, and level of noise, columns.* |

| 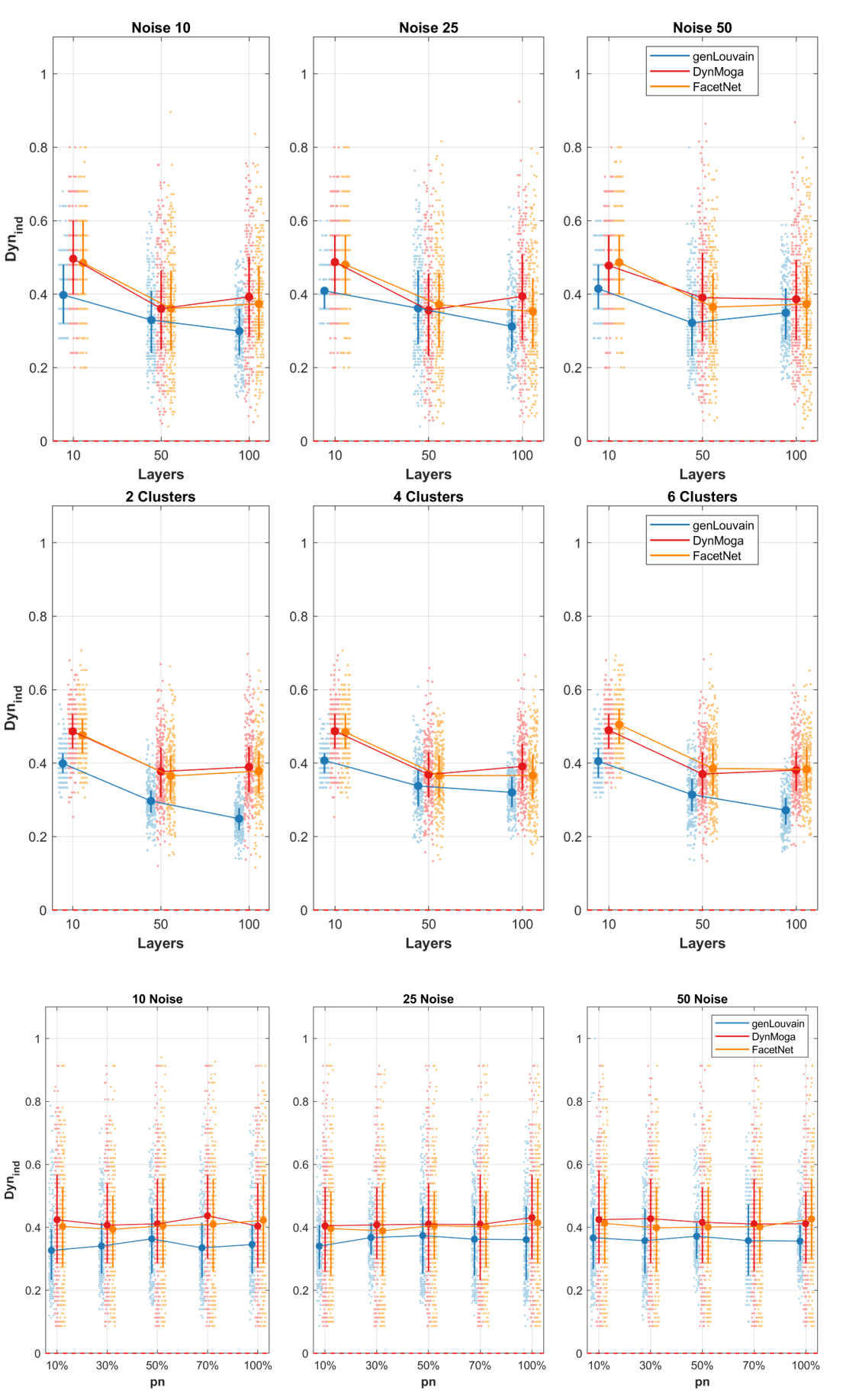 |
| --- |
| *Figure S16. Plot of means and standard deviations of* ${Dyn}_{ind}$ *in the comparative analysis on networks with D=0.1 evolving community structure. In the first row we report the dynamic of the algorithms, identified with different colors, with respect to the different levels of number of layers, x-axis, and percentage of noise, columns. In the second row we show the trend of the algorithms’ speed with respect to the number of layers, x-axis, and number of clusters, columns. In the third row we represent the* ${Dyn}_{ind}$ *mean values for each algorithm to varying of the factor pn, x-axis, and level of noise, columns.* |

# Comparative analysis on networks with evolving community structure and with increasing or decreasing clusters number

In this section we present the results of the comparative simulation studies (Section 2.2.2 of the main paper) in which we evaluated the algorithms performances in recovering a dynamic community structure with a variable number of clusters. Figures S13 reports the results obtained with an increasing number of clusters, while S14 reports the results for a decreasing number of clusters. We performed a repeated measures ANOVA with the factors *algorithm*=[genLouvain, ModStat, DynMoga, FacetNet], CN=[2, 4, 6] (*number of clusters*), no=[10%, 25%, 50%] (*noise level* ), nL=[2, 10, 50, 100] (*number of layers*), and pn=[10%, 30%, 50%, 70%, 100%] (*percentage of nodes changing allegiance to modules*). The multilayer networks have a density D=0.3; moving from the layer nL/2 to the layer (nL/2+1) the number of modules characterizing the community structure is increased (or decreased) of a unit. The results of these statistic tests are reported in Tables 7 and 8.

The trend of accuracies remains similar to those shown in the main analysis, with the algorithms all displaying a higher accuracy in non-noisy networks and with more than two clusters and low level of noise (no<25%). Again, genLouvain and FacetNet are the only algorithms whose performances depend on the number of layers in a proportional way. On the other hand, when the number of clusters of the network changes, the algorithm DynMOGA shows better performance than FacetNet. GenLouvain and ModStat are still the best algorithms, with the first outperforming the second in networks with increasing noise and layers. This time, the factor *pn* influences a little more the algorithms behavior. In network with noise *no*<50% all the algorithms are more accurate when the number of nodes that change cluster is high, meaning that they are more suitable to detect large changes, except for FacetNet who presents an opposite trend. This is true especially when the number of clusters in the networks is reduced.

| 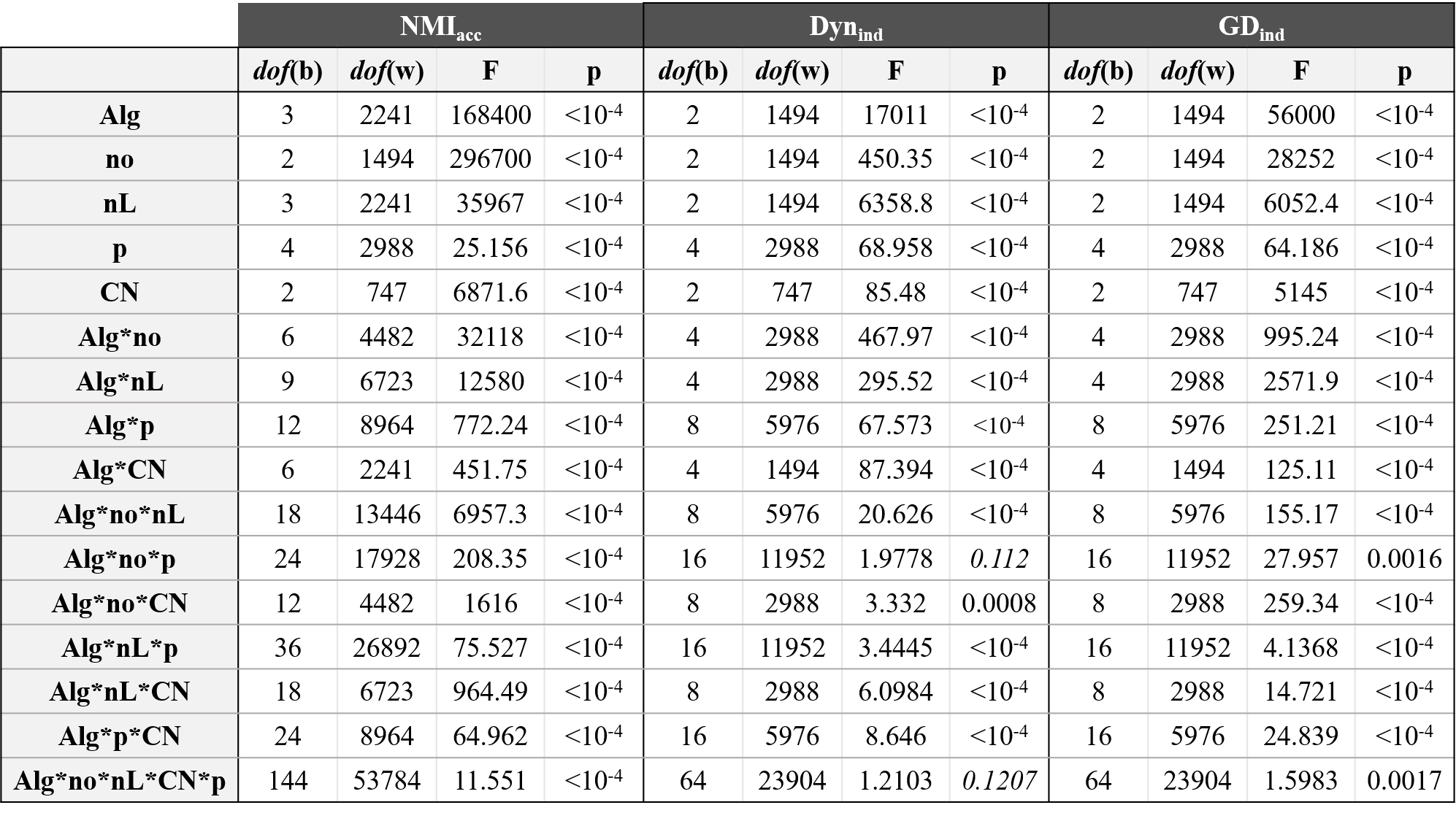 |
| --- |
| *Table S7. Results of the ANOVA test executed for the comparative analysis on networks with evolving community structure, increasing number of clusters, and graph density equal to 0.3. For each considered index (dependent variables of the test) we report the degrees of freedom (dof), F and p-values relative to single factors and the interactions among them.* |

| 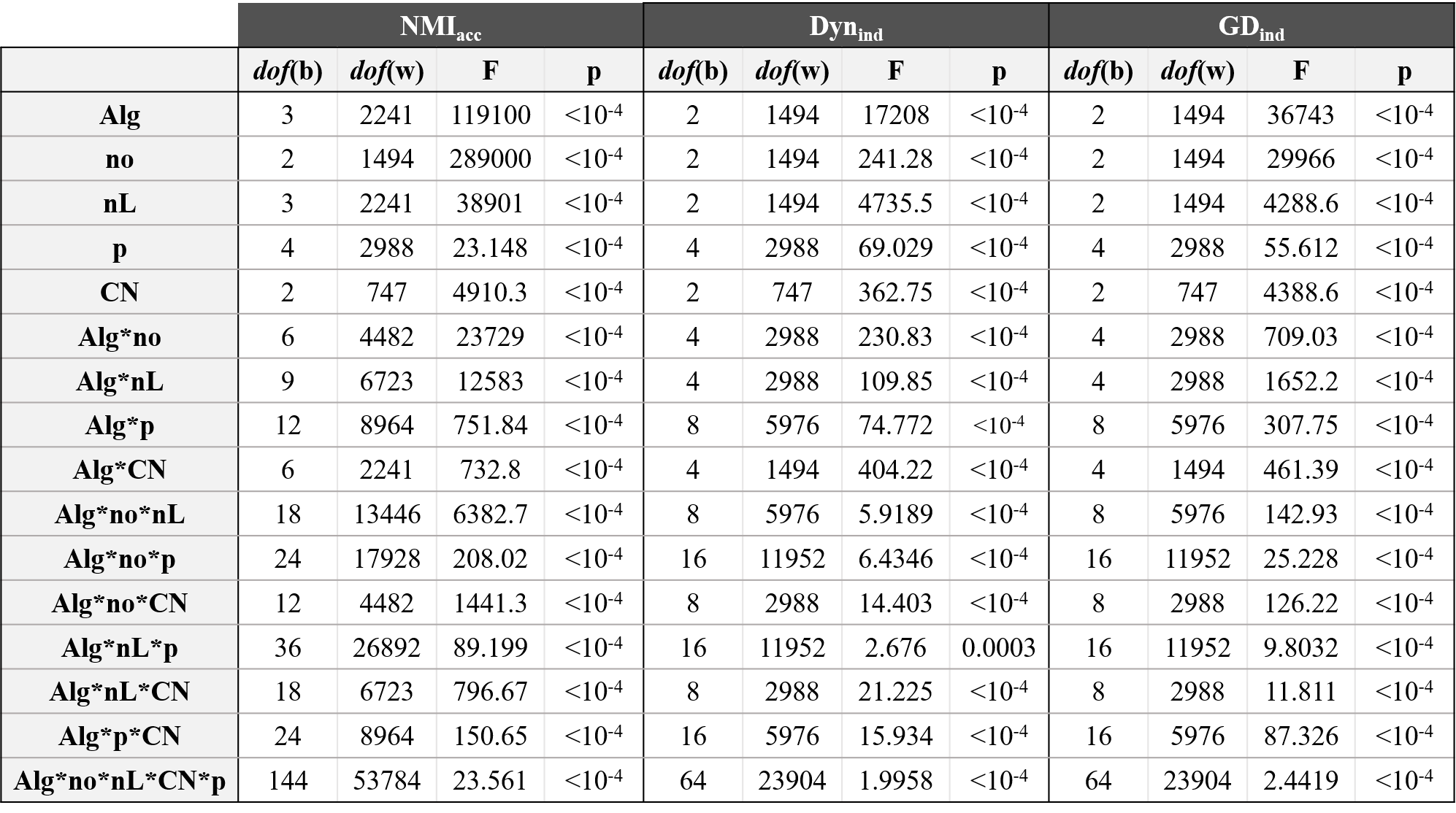 |
| --- |
| *Table S8. Results of the ANOVA test executed for the comparative analysis on networks with evolving community structure, decreasing number of clusters, and graph density equal to 0.3. For each considered index (dependent variables of the test) we report the degrees of freedom (dof), F and p-values relative to single factors and the interactions among them.* |

| 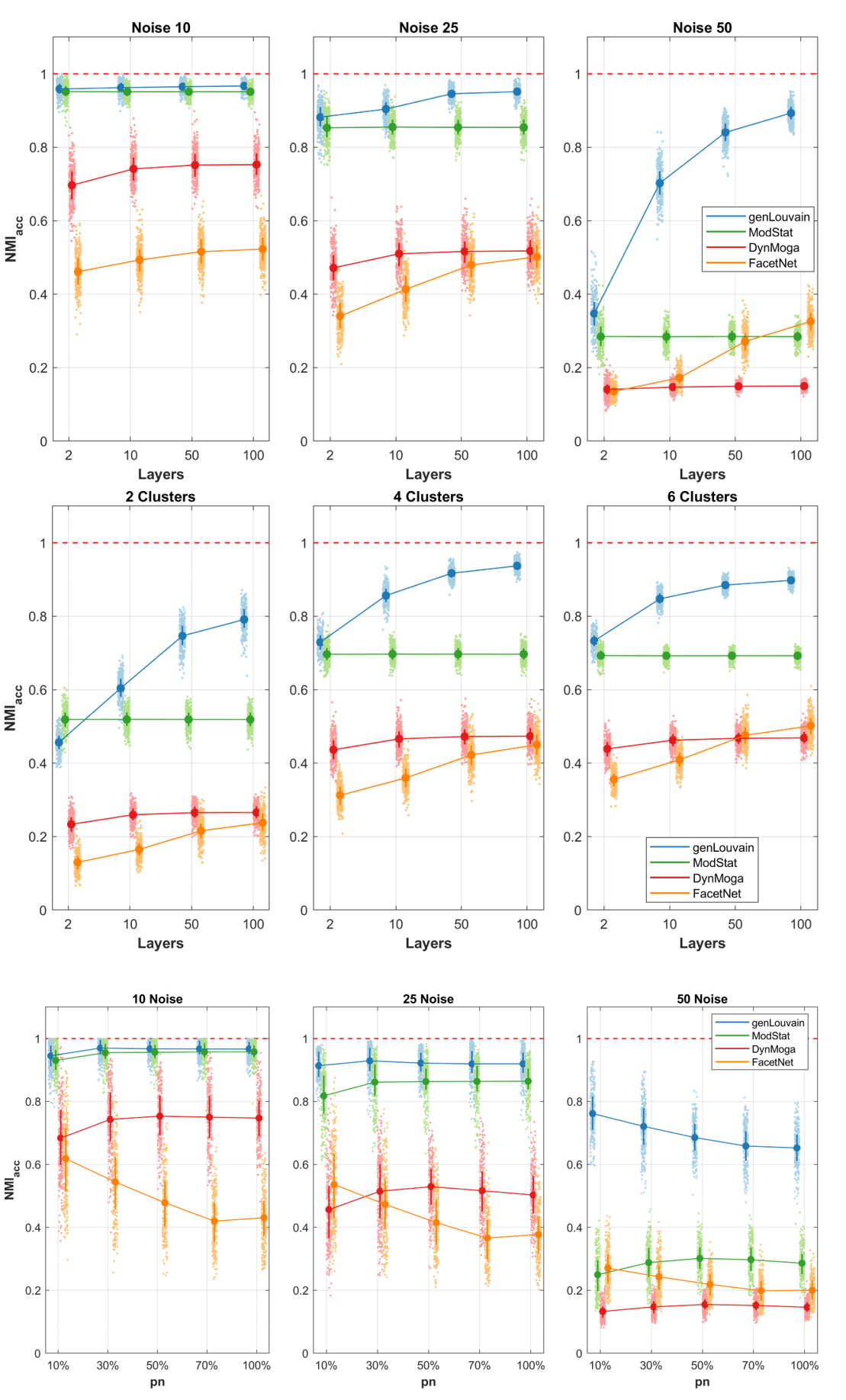 |
| --- |
| Figure S17. Plot of means and standard deviations of ${NMI}_{acc}$ in the comparative analysis on networks with evolving community structure with increasing number of clusters. In the first row we report the accuracy of the algorithms, identified with different colors, with respect to the different levels of number of layers, x-axis, and percentage of noise, columns. In the second row we show the trend of the algorithms’ accuracies with respect to the number of layers, x-axis, and number of clusters, columns. In the third row we represent the accuracies mean values for each algorithm to varying of the factor pn, x-axis, and level of noise, columns |

| 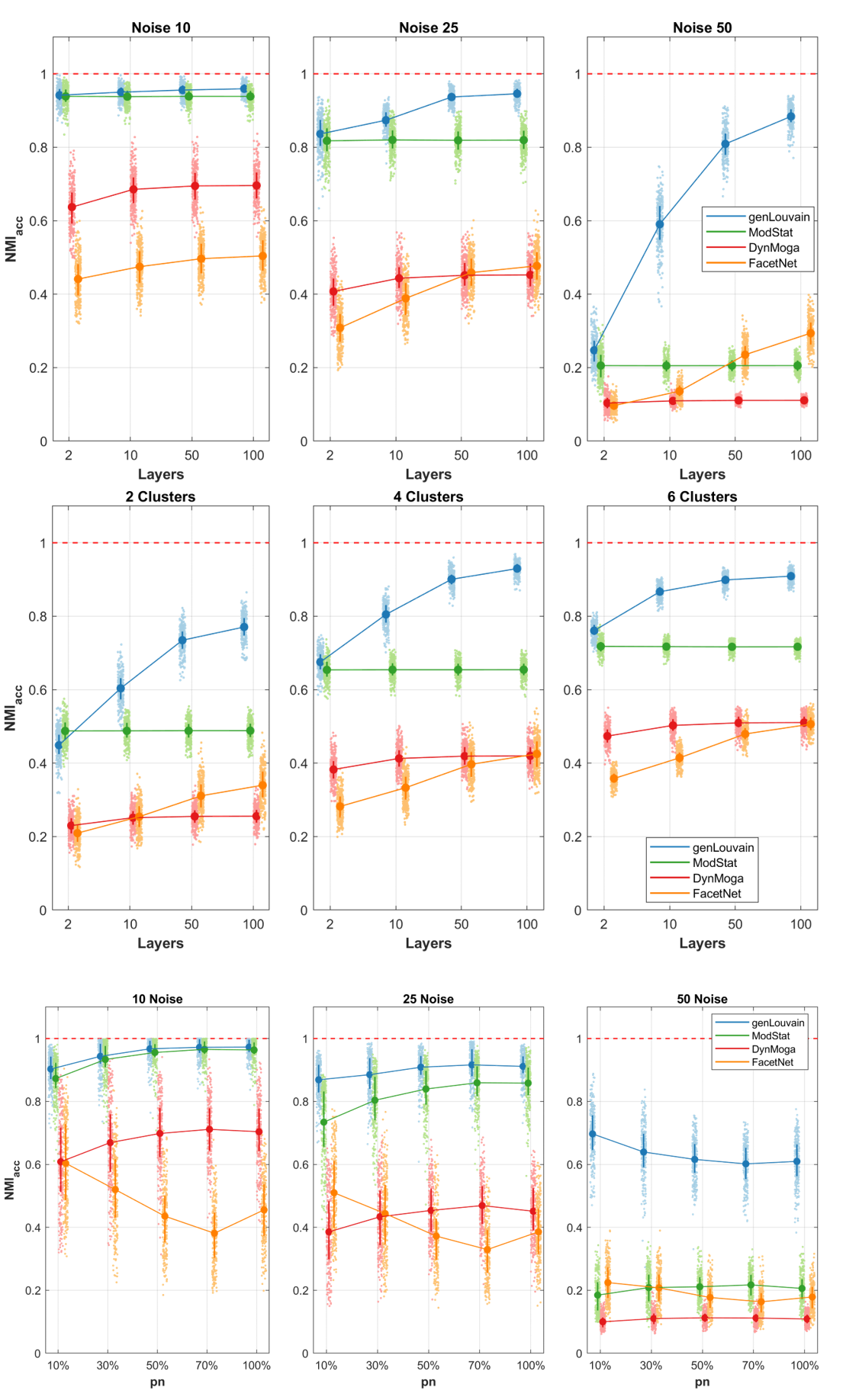 |
| --- |
| Figure S18. Plot of means and standard deviations of ${NMI}_{acc}$ in the comparative analysis on networks with evolving community structure with decreasing number of clusters. In the first row we report the accuracy of the algorithms, identified with different colors, with respect to the different levels of number of layers, x-axis, and percentage of noise, columns. In the second row we show the trend of the algorithms’ accuracies with respect to the number of layers, x-axis, and number of clusters, columns. In the third row we represent the accuracies mean values for each algorithm to varying of the factor pn, x-axis, and level of noise, columns |
